# Identification of Key Genes Associated with Tumor Microenvironment Infiltration and Survival in Gastric Adenocarcinoma via Bioinformatics Analysis

**DOI:** 10.3390/cancers16071280

**Published:** 2024-03-26

**Authors:** Georgios Konstantis, Georgia Tsaousi, Chryssa Pourzitaki, Stefan Kasper-Virchow, Gregor Zaun, Elisavet Kitsikidou, Moritz Passenberg, Vasilis Spyridon Tseriotis, Katharina Willuweit, Hartmut H. Schmidt, Jassin Rashidi-Alavijeh

**Affiliations:** 1 Clinical Pharmacology, Faculty of Medicine, School of Health Sciences, Aristotle University of Thessaloniki, 541 24 Thessaloniki, Greece; chpour@auth.gr (C.P.); tvasilis@auth.gr (V.S.T.); 2 Department of Gastroenterology, Hepatology and Transplant Medicine, Medical Faculty, University of Duisburg-Essen, 45141 Essen, Germany; 3 Department of Anesthesiology and ICU, Medical School, Aristotle University of Thessaloniki, 541 24 Thessaloniki, Greece; tsaousig@otenet.gr; 4 Department of Medical Oncology, West German Cancer Center, University Hospital Essen, Hufelandstr. 55, 45147 Essen, Germany; 5 Department of Internal Medicine, Evangelical Hospital Dusseldorf, 40217 Dusseldorf, Germany; elisavet.kitsikidou@evk-duesseldorf.de

**Keywords:** gastric adenocarcinoma, bioinformatics analysis, tumor microenvironment, gene expression omnibus, survival

## Abstract

**Simple Summary:**

This study aimed to pinpoint immune-related genes that show heightened activity in cancerous tissue and explore their correlation with cell infiltration in the tumor microenvironment using bioinformatics analysis. By examining gene expression from both stomach cancer and adjacent healthy tissues, we aimed to uncover their significance in cancer development and their impact on the body’s immune response. We identified several genes, including *FN1*, *COL1A2*, *THBS2*, *COL3A1*, *COL5A1*, and *BGN*, which appear to be associated with poorer outcomes for stomach cancer patients. These genes also demonstrate connections to specific immune cells within cancerous tissue. Understanding the role of these genes in the immune response to cancer could facilitate the development of novel treatments and enhance prognostic capabilities for individuals with stomach cancer.

**Abstract:**

Objective: Gastric carcinoma (GC) is the fifth most commonly diagnosed cancer and the third leading cause of cancer-related deaths globally. The tumor microenvironment plays a significant role in the pathogenesis, prognosis, and response to immunotherapy. However, the immune-related molecular mechanisms underlying GC remain elusive. Bioinformatics analysis of the gene expression of GC and paracancerous healthy tissues from the same patient was performed to identify the key genes and signaling pathways, as well as their correlation to the infiltration of the tumor microenvironment (TME) by various immune cells related to GC development. Methods: We employed GSE19826, a gene expression profile from the Gene Expression Omnibus (GEO), for our analysis. Functional enrichment analysis of Differentially Expressed Genes (DEGs) was conducted using the Gene Ontology and Kyoto Encyclopedia of Genes and Genomes database. Results: Cytoscape software facilitated the identification of nine hub DEGs, namely, *FN1*, *COL1A1*, *COL1A2*, *THBS2*, *COL3A1*, *COL5A1*, *APOE*, *SPP1*, and *BGN.* Various network analysis algorithms were applied to determine their high connectivity. Among these hub genes, *FN1*, *COL1A2*, *THBS2*, *COL3A1*, *COL5A1*, and *BGN* were found to be associated with a poor prognosis for GC patients. Subsequent analysis using the TIMER database revealed the infiltration status of the TME concerning the overexpression of these six genes. Specifically, the abovementioned genes demonstrated direct correlations with cancer-associated fibroblasts, M1 and M2 macrophages, myeloid-derived suppressor cells, and activated dendritic cells. Conclusion: Our findings suggest that the identified hub genes, particularly *BGN*, *FN1*, *COL1A2*, *THBS2*, *COL3A1*, and *COL5A1*, play crucial roles in GC prognosis and TME cell infiltration. This comprehensive analysis enhances our understanding of the molecular mechanisms underlying GC development and may contribute to the identification of potential therapeutic targets and prognostic markers for GC patients.

## 1. Introduction

Despite marked geographical and regional variations in the frequency of gastric cancer (GC), there has been a consistent decline in its incidence in Western countries in recent decades. Nevertheless, GC continues to hold its position as the fifth most commonly diagnosed cancer and ranks as the third leading cause of cancer-related deaths globally [1].

GC originates from the gastric epithelium and develops through a stepwise progression via precancerous conditions, such as chronic gastritis, advancing towards early carcinomas that infiltrate the mucosa and submucosa. GC has a multifactorial etiology. Endogenous risk factors, such as genetic predisposition, and exogenous factors, like chronic H. pylori-induced gastritis or the increased intake of nitrates, play pivotal roles in its development [2].

The treatment of gastric adenocarcinoma is stage-dependent and involves various multimodal approaches. In recent years, with advancements in endoscopic intervention capabilities, endoscopic submucosal dissection (ESD) and endoscopic mucosal resection (EMR) have emerged as the preferred methods for treating early stage GC, while surgical intervention remains the therapy of choice for more advanced cases [3,4]. Despite progress in understanding the pathogenesis and pathophysiology of GC, perioperative therapy primarily relies on systemic chemotherapy regimens, such as fluorouracil, oxaliplatin, docetaxel, and leucovorin (FLOT) or epirubicin, cisplatin, and capecitabine (ECX), leading to considerable side effects. While neoadjuvant and adjuvant chemotherapy represent essential approaches, immunotherapy plays a limited role in GC treatment compared to many other cancer types [4]. As an example, recent investigations propose that the combination of pembrolizumab and chemotherapy may represent a therapeutic approach for individuals diagnosed with locally advanced or metastatic HER2-negative gastric or gastroesophageal junction adenocarcinoma [5]. Additionally, nivolumab has exhibited superior overall survival when administered alongside chemotherapy compared to sole chemotherapy in previously untreated patients with an advanced gastric, gastroesophageal junction, or esophageal adenocarcinoma [6].

The term tumor microenvironment (TME) refers to the intricate relationships between host cells and malignant cells that develop as a result of tumor cell infiltration [7]. Tumor cells induce substantial molecular, cellular, and physical alterations within the tissues they invade. While the composition of the TME differs among various types of tumors, common characteristics encompass immune cells, stromal cells, blood vessels, and the extracellular matrix (ECM) [8]. The heterogeneity of the TME in GC, particularly with regard to the complexity of the immunological aspects [9], contributes to the lack of widespread acceptance and success of immunotherapy, such as in non-small cell lung cancer or renal cell carcinoma. Therefore, further exploration of the TME and the identification of the key genes related to immune infiltration has garnered significant attention and may serve as a crucial step toward decoding this complexity and the development of new therapies. Through the analysis of the TME, it is hoped that, in the future, it will be possible to predict which GC patients will benefit from immune–oncology treatments and which will not.

Microarray technology, coupled with bioinformatics analysis, has been extensively employed to scrutinize genetic variations across genome sequencing [10]. Despite numerous studies employing these methodologies to explore the clinical relevance of TME infiltrates [11,12], a comprehensive understanding of the diverse array of cells infiltrating the TME remains elusive. Notably, a recent bioinformatics investigation unveiled Stromal-Related Gene Signatures that are intricately linked to macrophage infiltration [13].

The aim of our study was to identify immune-related genes that are overexpressed in cancerous tissue and investigate the relationship of these genes with cell infiltration in the TME through bioinformatics analysis. Subsequently, we determined which of these genes play a significant role in the pathogenesis and progression of GC, potentially serving as targets for pharmaceutical therapies. In our study, we utilized GSE19826 [14], a gene expression profile retrieved from the Gene Expression Omnibus (GEO), for analysis. Subsequently, we conducted a functional enrichment analysis of Differentially Expressed Genes (DEGs), using both the Gene Ontology (GO) and the Kyoto Encyclopedia of Genes and Genomes (KEGG) database. We identified hub DEGs demonstrating high connectivity. Following this, we delved into assessing the infiltration status of immune cells in GC using the Tumor Immune Evaluation Resource (TIMER) [15]. Finally, we attempted to establish correlations between these hub DEGs and immune cells, revealing pertinent associations in our research.

## 2. Materials and Methods

### 2.1. Microarray Data

The expression profiles of genes associated with gastric adenocarcinoma were obtained from the GEO database (https://www.ncbi.nlm.nih.gov/geo/, accessed on 1 December 2023). One gene expression profile was derived from human gastric adenocarcinoma samples. The GSE19826 series [14] includes 12 samples of GC human tissue and 12 samples of corresponding non-cancerous human tissue from the same patients, along with 3 samples from healthy individuals serving as controls. We utilized the 12 cancerous and 12 non-cancerous samples from the same patients for our analysis. The populations of cancer tissues at each TNM stage were homogenous. Based on the annotation information from the platform, probes were converted into corresponding gene symbols. Additional information regarding the basic characteristics of the patients can be directly acquired from the gene expression profile [14].

### 2.2. Identification of DEGs between Normal and GC Tissue

The GEO2R tool, available within the GEO series (https://www.ncbi.nlm.nih.gov/geo/geo2r/, accessed on 1 December 2023) [16], was employed to discern DEGs between non-cancerous tissue and GC tissue samples. Genes lacking corresponding gene symbols and those represented by multiple probes were excluded independently. A threshold of |Log2FC| > 1.5 and an adjusted *p* < 0.05 were applied as criteria for statistical significance.

### 2.3. Functional Enrichment Analyses of the GC-Related DEGs

In order to explore the biological roles of the identified DEGs within cellular components (CCs), molecular functions (MFs), and biological processes (BPs), the ‘pathfindR’ package [17] was employed to conduct enrichment analyses using GO and the KEGG pathways [18]. The ‘pathfindR’ package was chosen on the basis that it does not solely rely on direct enrichment analysis and thus provides a more comprehensive perspective. Depending solely on a list of significant genes might not provide sufficient information to fully comprehend the underlying disease mechanisms.

### 2.4. Construction of PPI Network and Identification of Hub DEGs

Furthermore, the GC-associated DEGs identified through the aforementioned methods underwent analysis in the STRING online analysis tool (http://string-db.org, Version: 11.5, accessed on 1 December 2023) [19]. This step was aimed at predicting potential protein interactions among the encoded proteins, utilizing a medium confidence score (>0.4). Utilizing the outcomes obtained from the STRING analysis, a PPI network involving these genes was constructed using the Cytoscape software platform (version: 3.7.10) [20]. To pinpoint the top 10 hub DEGs associated with this network, the ‘cytoHubba’ plugin software in Cytoscape employed a hybrid computation, integrating EPC, Degree, MNC, MCC, and Bottleneck algorithms. This methodology facilitated the further identification of the top 10 hub DEGs.

### 2.5. Prognostic Value of Hub DEGs as Biomarkers in GC

The prognostic value of the identified top 10 gene hubs was assessed using Kaplan–Meier curves from OncoLnc [21]. OncoLnc encompasses survival information for 8647 individuals across 21 cancer investigations conducted by The Cancer Genome Atlas. These curves were instrumental in evaluating the prognostic efficacy for patients diagnosed with GC, enabling differentiation between those with high and low expression levels of the specified genes.

### 2.6. Correlation Analyses between Hub DEGs and Infiltrating Immune Cells

The potential associations between the hub DEGs and infiltrating immune cells within the TME were explored using Spearman correlation analysis performed in R [22] using TIMER [15]. TIMER is an open-source server used for a comprehensive analysis of tumor-infiltrating cells in various cancers.

### 2.7. Expression of Hub Genes in Normal or GC Tissue

The expression analysis of hub genes between normal or GC samples was conducted by Student’s *t*-test. A *p*-value < 0.05 was considered significant.

## 3. Results

### 3.1. Identification of DEGs between Normal and GC Tissue

**Figure 1:**
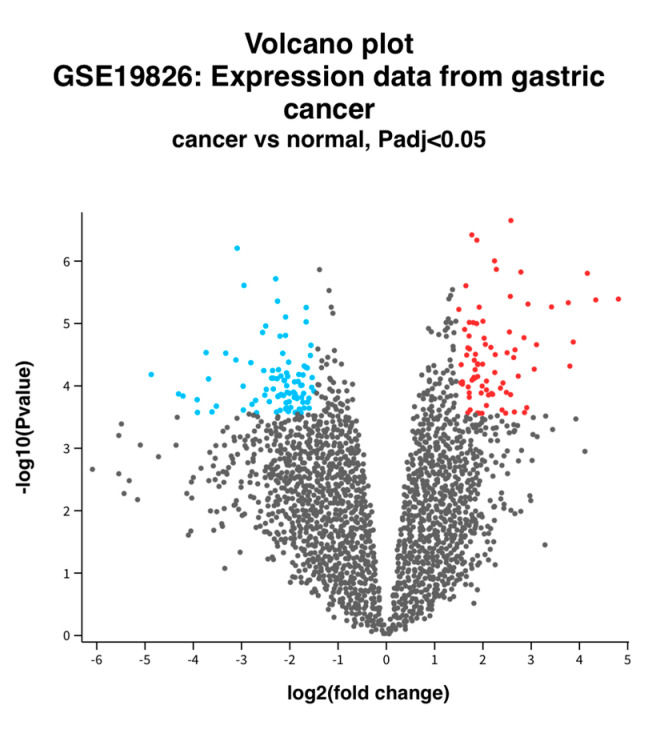
Volcano plot of DEGs between normal and GC tissue. Volcano plot depicting the up-regulated and down-regulated genes. Red indicates higher expression, while blue indicates lower expression. Log2FC > 1.5 and an adjusted *p*-value of <0.05 were used.

In GSE19826, a total of 153 DEGs were confirmed in the cancer tissue, including 91 down-regulated genes and 62 up-regulated genes (Figure 1). The genes are more precisely listed in Supplementary Table S1.

### 3.2. Functional Enrichment Analyses

**Figure 2:**
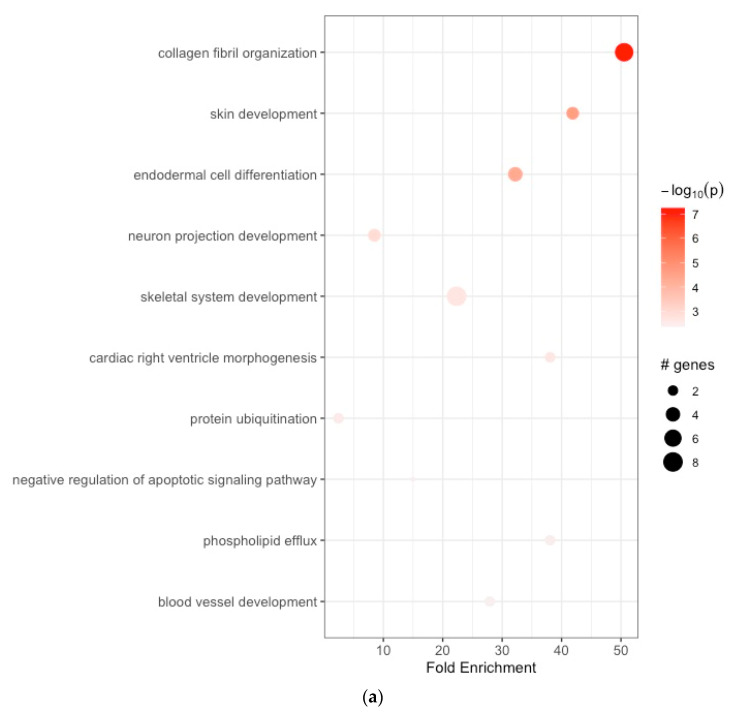

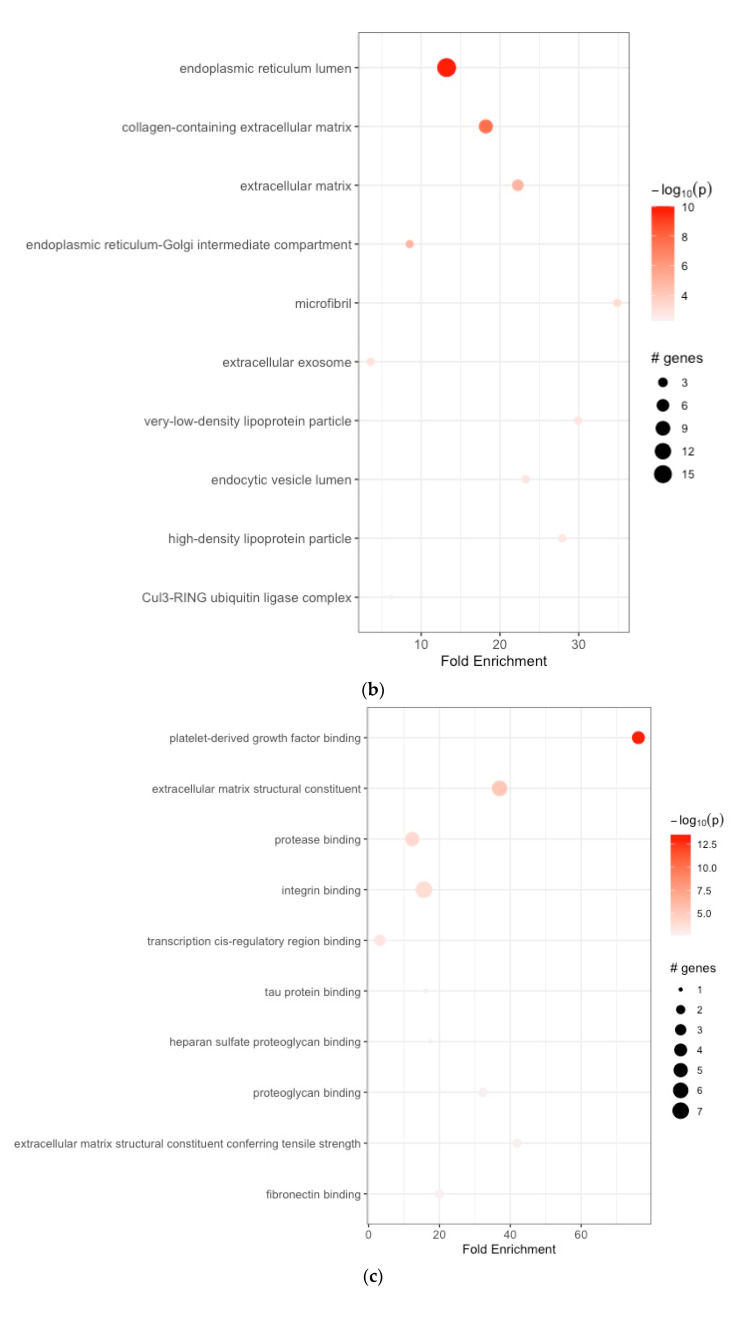

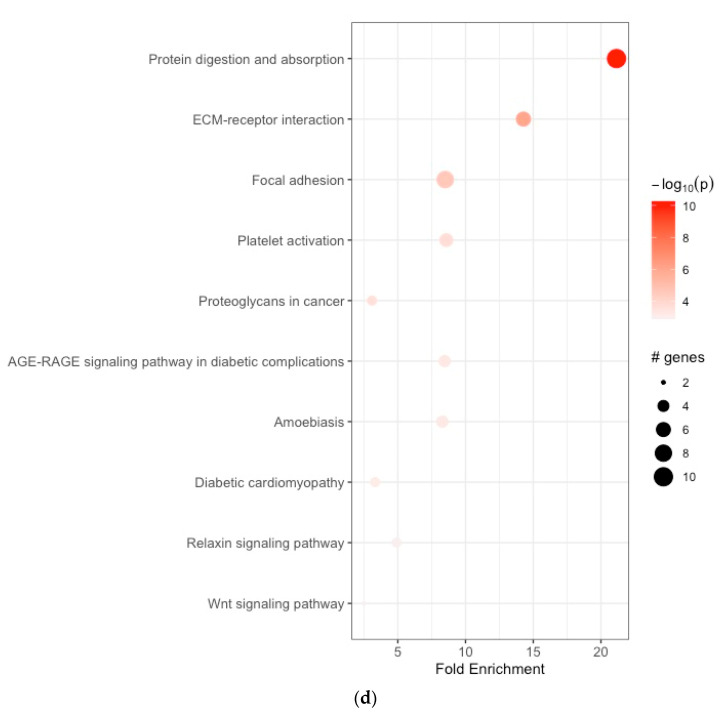
(**a**) Enrichment analysis of gastric cancer-related DEGs. GO analysis: enriched biological processes; (**b**) functional enrichment analyses GO: cellular components; (**c**) functional enrichment analyses GO: molecular functions; (**d**) KEGG pathway enrichment analysis.

The enriched BPs included collagen fibril organization, skin development, endodermal cell differentiation, neuron projection development, skeletal system development, cardiac right ventricle morphogenesis, protein ubiquitination, negative regulation of the apoptotic signaling pathway, phospholipid efflux, and blood vessel development (Figure 2a, Table 1a). The CCs were endoplasmic reticulum lumen, collagen-containing extracellular matrix, extracellular matrix, endoplasmic reticulum–Golgi intermediate compartment, microfibril, extracellular exosome, very-low-density lipoprotein particle, endocytic vesicle lumen, high-density lipoprotein particle, and Cul3-RING ubiquitin ligase complex (Figure 2b, Table 1b). The enriched MFs included platelet-derived growth factor binding, extracellular matrix structural constituent, protease binding, integrin binding, transcription cis-regulatory region binding, tau protein binding, heparan sulfate proteoglycan binding, proteoglycan binding, extracellular matrix structural constituent conferring tensile strength, and fibronectin binding (Figure 2c, Table 1c). The KEGG pathway analysis revealed enrichment in protein digestion and absorption, ECM–receptor interaction, focal adhesion, platelet activation, proteoglycans in cancer, the AGE-RAGE signaling pathway in diabetic complications, amoebiasis, diabetic cardiomyopathy, the relaxin signaling pathway, and the Wnt signaling pathway (Figure 2d, Table 1d).

### 3.3. Construction of PPI Network and Identification of Hub DEGs

**Figure 3:**
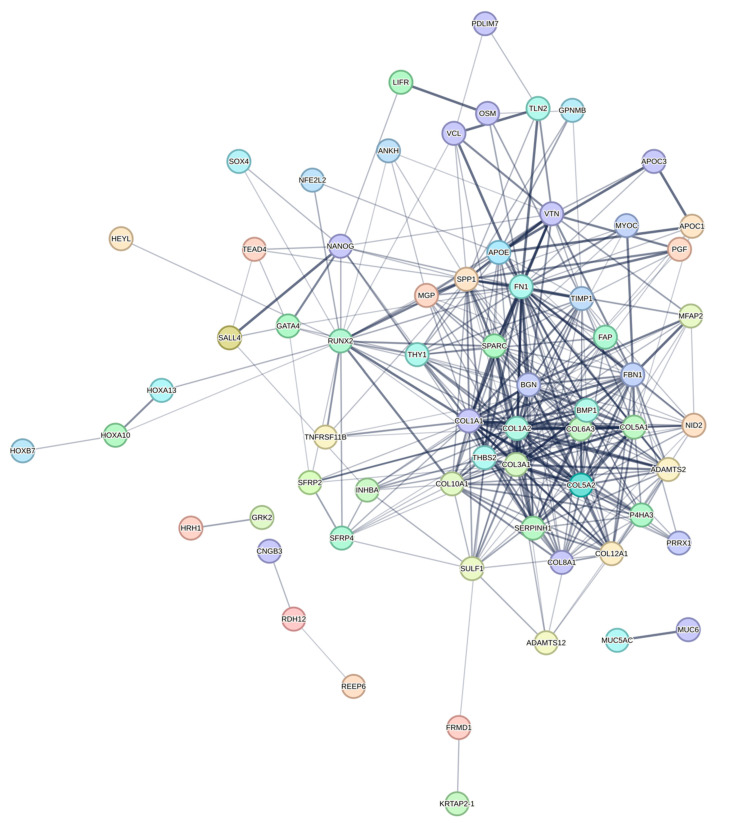
The PPI network of differentially expressed GC-related genes.

**Figure 4:**
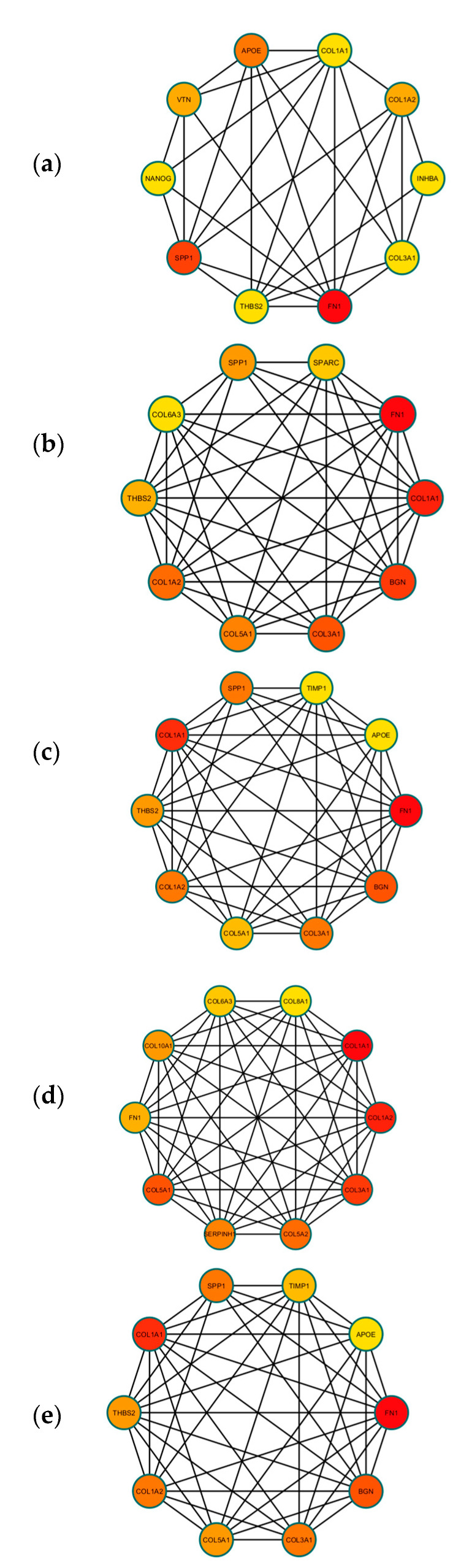
(**a**) The top 10 hub DEGS with the highest connectivity extracted by Bottleneck (color depth for ranking of hub DEGs). (**b**) The top 10 hub DEGS with the highest connectivity extracted by EPC (color depth for ranking of hub DEGs). (**c**) The top 10 hub DEGS with the highest connectivity extracted by Degree (color depth for ranking of hub DEGs). (**d**) The top 10 hub DEGS with the highest connectivity extracted by MCC (color depth for ranking of hub DEGs). (**e**) The top 10 hub DEGS with the highest connectivity extracted by MNC (color depth for ranking of hub DEGs).

**Figure 5:**
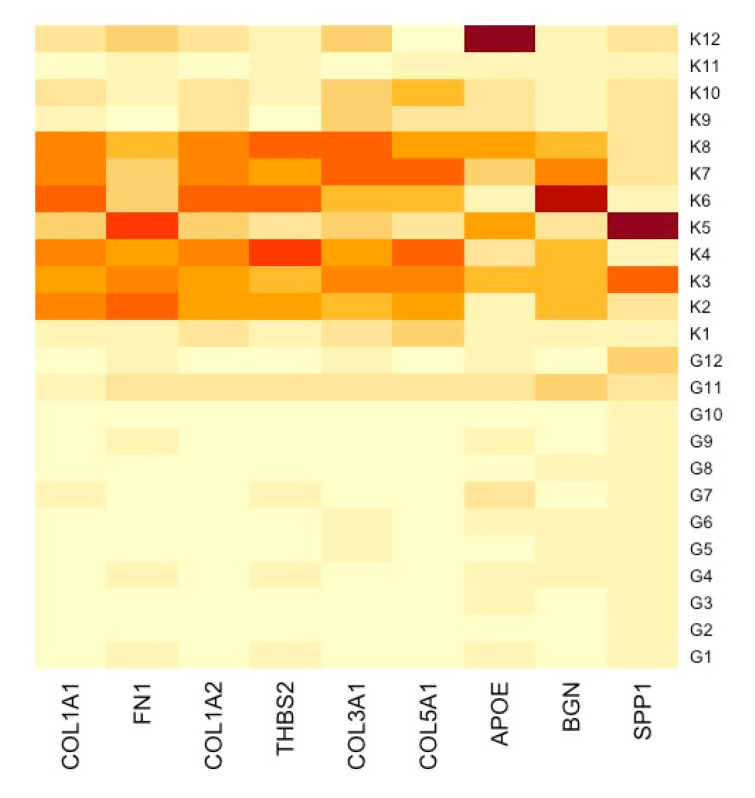
Heatmap showcasing distinctly the gene expression alterations between GC samples and their respective normal tissue counterparts. K represents normal gastric tissue, and G represents GC tissue.

**Figure 6:**
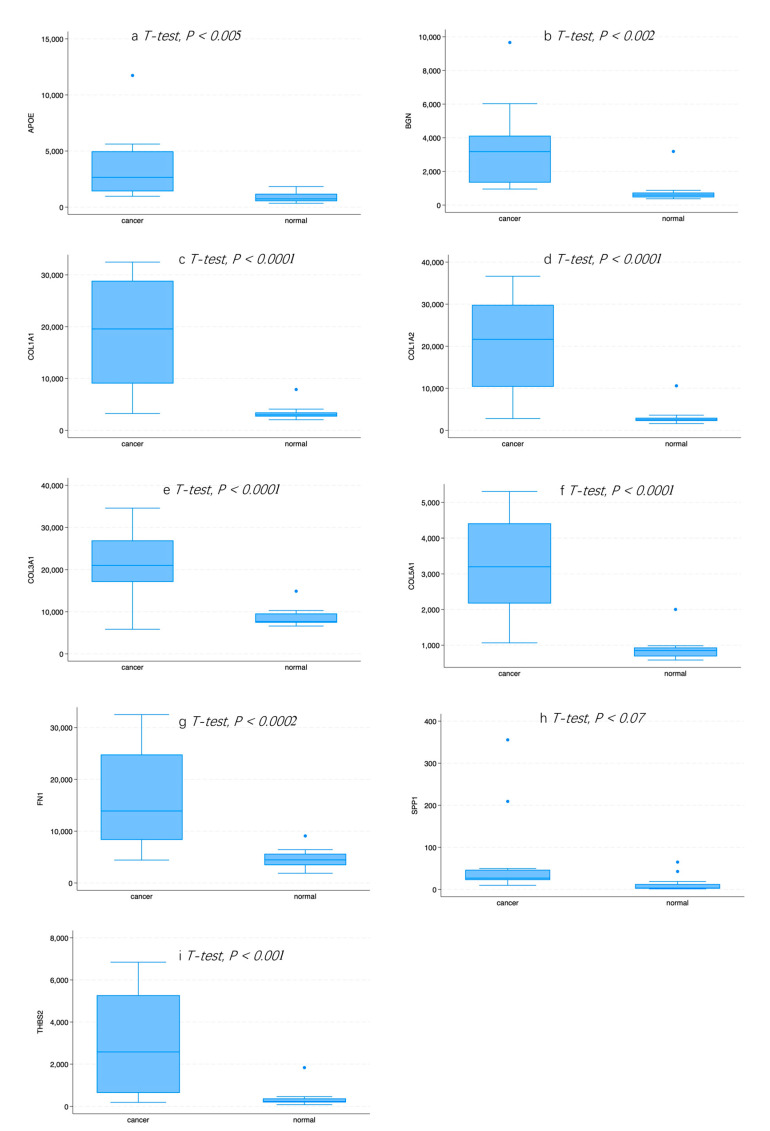
(**a**) The expression pattern of APOE in normal and GC tissue. (**b**) The expression pattern of BGN in normal and GC tissue. (**c**) The expression pattern of COL1A1 in normal and GC tissue. (**d**) The expression pattern of COL1A2 in normal and GC tissue. (**e**) The expression pattern of COL3A1 in normal and GC tissue. (**f**) The expression pattern of COL5A1 in normal and GC tissue. (**g**) The expression pattern of FN1 in normal and GC tissue. (**h**) The expression pattern of SPP1 in normal and GC tissue. (**i**) The expression pattern of THBS2 in normal and GC tissue.

A PPI network was created using the DEGs associated with GC (Figure 3). Concurrently, the identification of highly connected DEGs was performed through the utilization of five computational methods within the Cytoscape software platform (Figure 4a–e). This analysis involved the application of EPC, Degree, MNC, MCC, and Bottleneck algorithms (Supplementary Table S2). Following this, nine hub genes (*FN1*, *COL1A1*, *COL1A2*, *THBS2*, *COL3A1*, *COL5A1*, *APOE*, *SPP1*, and *BGN*) were identified from the overlap among the top hub DEGs obtained through the application of the five methods. The differences in expression levels of the previously listed genes between GC and normal tissues were visualized using a heatmap (Figure 5). Additionally, we obtained the variance comparison between GC and normal tissues (Figure 6a–i).

### 3.4. Survival Analysis of Hub DEGs in GC

**Figure 7:**
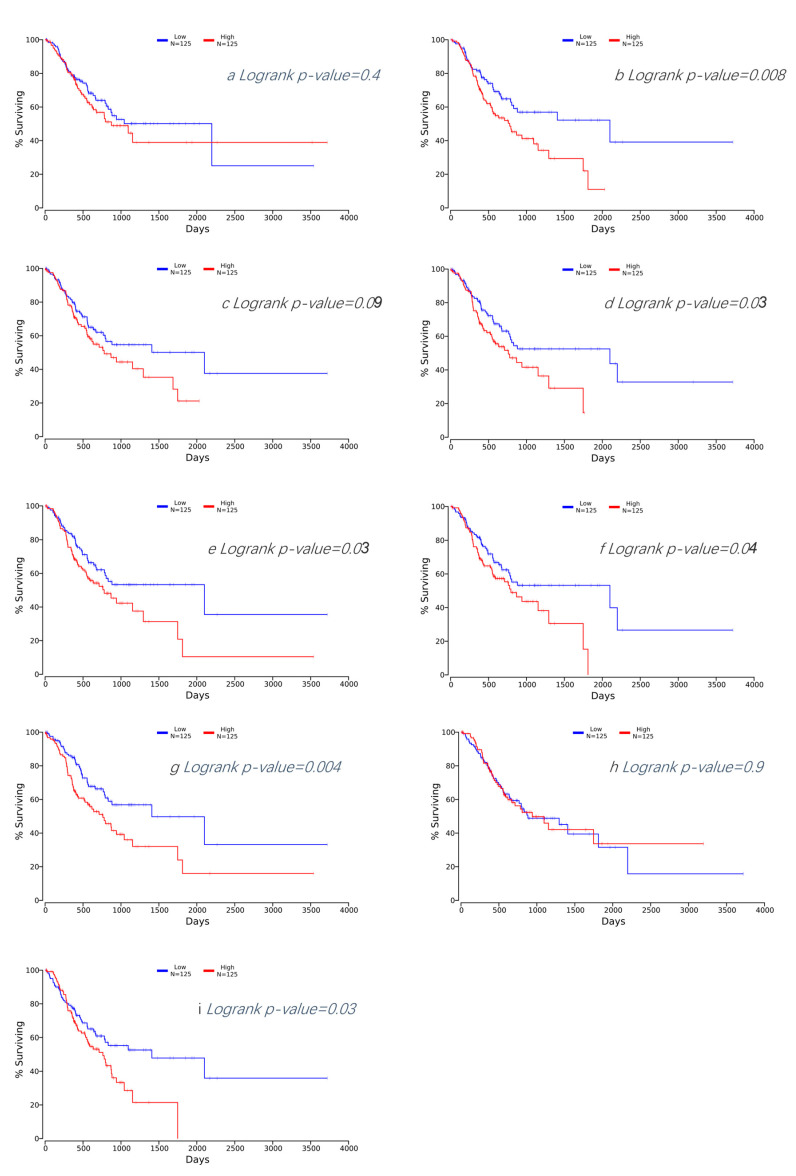
(**a**) Kaplan–Meier plot for APOE in GC. (**b**) Kaplan–Meier plot for BGN in GC. (**c**) Kaplan–Meier plot for COL1A1 in GC. (**d**) Kaplan–Meier plot for COL1A2 in GC. (**e**) Kaplan–Meier plot for COL3A1 in GC. (**f**) Kaplan–Meier plot for COL5A1 in GC. (**g**) Kaplan–Meier plot for FN1 in GC. (**h**) Kaplan–Meier plot for SPP1 in GC. (**i**) Kaplan–Meier plot for THBS2 in GC.

Our results indicate that patients with an elevated expression of six hub DEGs, screened out by the five methods mentioned above, were associated with a poor survival rate. The corresponding Kaplan–Meier curves can be seen in Figure 7a–i. Patients with an increased expression of *FN1*, *COL1A2*, *THBS2*, *COL3A1*, *COL5A1*, and *BGN* showed a poorer prognosis.

### 3.5. TME Evaluation in GC

**Figure 8:**
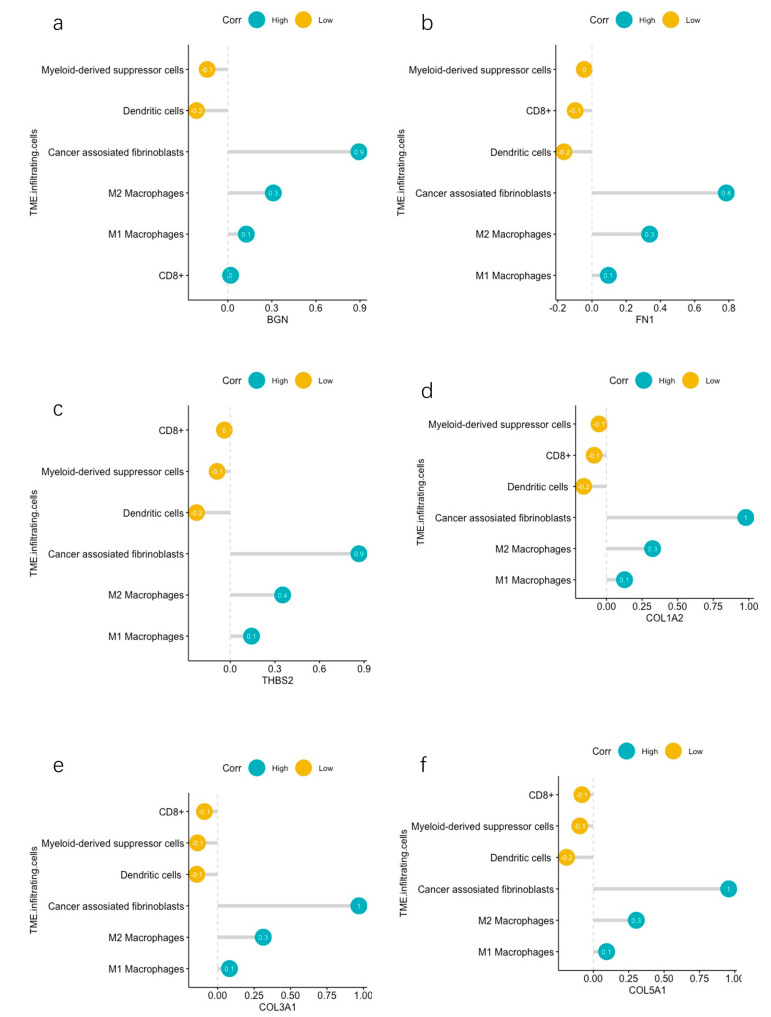
(**a**) Correlations between *BGN* and infiltrating immune cells and CAFs. (**b**) Correlations between *FN1* and infiltrating immune cells and CAFs. (**c**) Correlations between *THBS2* and infiltrating immune cells and CAFs. (**d**) Correlations between *COL1A2* and infiltrating immune cells and CAFs. (**e**) Correlations between *COL3A1* and infiltrating immune cells and CAFs. (**f**) Correlations between *COL5A1* and infiltrating immune cells and CAFs.

We investigated the correlation between the expression of the aforementioned selected genes, which were associated with survival, and the infiltration of cancer-associated fibroblasts, CD8^+^ activated dendritic cells, macrophages, and myeloid-derived suppressor cells (MDSCs). *BGN* demonstrated a direct correlation with cancer-associated fibroblasts, as well as M1 and M2 macrophages. Conversely, it exhibited an inverse relationship with MDSCs and activated dendritic cells (Figure 8a). *FN1* exhibited a positive connection with cancer-associated fibroblasts and M2 macrophages while demonstrating a negative correlation with activated dendritic cells (Figure 8b). *THBS2* showcased a positive correlation with M1 and M2 macrophages and cancer-associated fibroblasts. In contrast, it showcased a negative correlation with activated dendritic cells (Figure 8c). *COL1A2* displayed a positive association with M1 and M2 macrophages and cancer-associated fibroblasts. Conversely, it exhibited a negative association with activated dendritic cells (Figure 8d). *COL3A1* demonstrated a positive correlation with M2 macrophages and cancer-associated fibroblasts. It also showcased a negative correlation with activated dendritic cells and MDSCs (Figure 8e*). COL5A1* depicted a positive association with M2 macrophages and cancer-associated fibroblasts. Simultaneously, it portrayed a negative association with activated dendritic cells (Figure 8f). The Pearson correlation curves can be seen in the Supplementary Figures.

## 4. Discussion

The aim of this study was to identify the key genes involved in the pathogenesis and treatment of gastric adenocarcinoma, particularly linking these genes with components of the TME. This endeavor aimed to ascertain whether these genes and their corresponding proteins could serve as therapeutic targets through immunotherapy. Our study involved analyzing a publicly available GEO dataset to identify DEGs between human GC tissues and non-tumor tissues. A pool of 153 DEGs was identified as potential candidate biomarkers.

The analysis of the enrichment function unveiled the mechanisms driving the DEGs. Gene Ontology profiling highlighted substantial associations of DEGs with CCs, including the endoplasmic reticulum lumen, endoplasmic reticulum–Golgi intermediate compartment, and processes within the extracellular matrix, such as collagen fibril organization and the structural constitution of the collagen-containing extracellular matrix, implying that these genes may play a role in the progression of GC. Furthermore, DEGs were found to be involved in specific biological processes, such as endodermal cell differentiation, neuron projection development, and skeletal system development, as well as functions like platelet-derived growth factor binding, protease binding, integrin binding, and skin development. Additionally, our Kyoto Encyclopedia of Genes and Genomes analysis unveiled their involvement in pathways such as protein digestion and absorption, ECM–receptor interaction, focal adhesion, platelet activation, proteoglycans in cancer, the AGE-RAGE signaling pathway in diabetic complications, amoebiasis, diabetic cardiomyopathy, the relaxin signaling pathway, and the Wnt signaling pathway. Specifically, *COL1A1*, *COL1A2*, *COL6A3*, *THBS2*, *FN1*, and *SPP1* were found to be associated with both focal adhesion and ECM–receptor interaction pathways. Cell–matrix adhesions are crucial for fundamental biological processes. At these focal adhesions, actin filaments connect to integrin receptors through a complex network of junctional plaque proteins [23,24]. Interactions between cells and the extracellular matrix involve transmembrane molecules like integrins and possibly proteoglycans, CD36, or other cell surface-related components. These interactions directly or indirectly govern cellular activities, such as adhesion, migration, differentiation, proliferation, and apoptosis [23,24]. Signaling pathways involving proteins like FAK initiate downstream effects that trigger the reorganization of the actin cytoskeleton. Notably, the active form of FAK, pFAK, has been linked to a poorer prognosis in patients diagnosed with GC [25]. These findings highlight the emerging significance of the ECM in the pathogenesis and progress of GC.

Another point to emphasize is the significant enrichment of the platelet activation pathway in our KEGG analysis. The genes that were up-regulated were *TLN2*, *COL1A1*, *COL1A2*, *COL3A1*, and *PLA2G4C*. Platelet activation and cancer share extensive and intricate interactions [26,27]. These interactions are not unidirectional: the TME can activate platelets, leading to an increased risk of thrombosis and a worse prognosis [28], while conversely, platelets themselves are associated with promoting tumor progression. Specifically, when activated, they release transforming growth factor beta (TGF-β), vascular endothelial growth factor (VEGF), and platelet-derived growth factor (PDGF) [29], which can facilitate angiogenesis and tumoral neovascularization. *COL1A1*, *COL1A2*, and *COL3A1* belong to the group known as collagen fibers and have the potential to trigger the activation of GPV1. Consequently, this leads to platelet activation and their degranulation. PLA2G4C encodes the enzyme phospholipase A2γ, which hydrolyzes glycerophospholipids to produce free fatty acids and lysophospholipids [30]. Delving into the intricacies of platelet activation within the context of GC progression could potentially yield crucial insights into enhancing patient outcomes and augmenting the effectiveness of immunotherapeutic interventions.

Additionally, we discovered nine major hub genes using the STRING database to establish the PPI network and by employing five computational methods within the Cytoscape software platform. These genes include *FN1*, *COL1A1*, *COL1A2*, *THBS2*, *COL3A1*, *COL5A1*, *APOE*, *SPP1*, and *BGN*. Further survival analysis of these genes indicates that six (*FN1*, *COL3A1*, *COL5A1*, *BGN*, *THBS2*, and *COL1A2*) out of these nine up-regulated genes were significantly associated with an unfavorable prognosis for patients with GC.

The TME is a sophisticated milieu consisting of fibroblasts, endothelial cells, and diverse immune cells [31]. The cells within the TME actively support and stimulate other cell types and extracellular structures. This process fosters an environment that enables tumor cells to evade host immune surveillance and develop increased resistance to cancer therapy. Certain components of the TME deserve special attention, and our study focused on exploring specific aspects. There is mounting evidence showcasing the functional diversity of cancer-associated fibroblasts (CAFs) in studies on gastric adenocarcinoma. Typically, the definition of CAFs serves as an overarching term referring to a heterogeneous group of activated stromal cells. These cells exhibit functions that are distinct and set them apart from those of normal fibroblasts (NFs) [32]. Generally, CAFs play a pivotal role in the tumorigenesis process and cancer progression by releasing various ECM proteins and regulatory molecules [33]. Several studies have demonstrated the capability of CAFs to promote cancer invasion and migration through close interactions with tumor cells [33,34,35]. Additionally, CAFs regulate angiogenesis, immune suppression, and foster chemoresistance in cancer cells [33,36]. It has been demonstrated that CAF infiltration is linked to an immunosuppressive microenvironment and poorer survival outcomes in GC [37]. Cytotoxic CD8^+^ T cells are pivotal effectors in the anti-cancer immune response. They constitute a critical element of cancer immunotherapy owing to their pleiotropic effects. Solid tumors can be categorized into ‘cold’ and ‘hot’ tumors [38]. This categorization relies, in part, on the quantity of infiltrating T cells, where a ‘hot’ tumor refers to a higher number of CD8^+^ T cells, whereas a ‘cold’ tumor represents the opposite [39]. They have the ability to migrate into the TME, and once differentiated into cytotoxic cells, they can exert cytotoxic effects against cancer cells [40,41]. Additionally, numerous studies have associated increased CD8^+^ T cell infiltration in the cancer microenvironment with a better response to immunotherapy [42]. Macrophages are among some of the most prevalent cell types found in the cancer microenvironment [43]. These cells, known as tumor-associated macrophages (TAMs) [44], are broadly classified into two main subtypes: M1-like and M2-like macrophages [45]. This categorization is based on distinct genetic and functional characteristics that align with those observed in normal macrophages. M1 macrophages respond to cytokines and bacterial lipopolysaccharides, which trigger their activation. Consequently, they release various molecules, such as nitric oxide synthase, reactive oxygen species, and the cytokine IL-12, inducing damage to target cells [46,47]. Essentially, these cells exert an anti-cancer effect by eliminating tumor cells [48]. Conversely, the differentiation of macrophages into the M2 phenotype is influenced by factors including CSF-1, IL-4, IL-13, and IL-10 [46,47]. These cells contribute to anti-inflammatory responses and support tumor growth by suppressing the immune system [48]. Under normal conditions, immature myeloid cells migrate from the bone marrow to peripheral organs, where they undergo rapid maturation into macrophages, dendritic cells, or granulocytes [49]. However, within the TME, various factors disrupt the usual maturation process of these immature myeloid cells, leading to the development of an immunosuppressive phenotype [50]. These myeloid-derived suppressor cells (MDSCs) demonstrate a pivotal role in hindering adaptive antitumor immunity by impeding T-cell activation and function while also facilitating the recruitment and stimulation of T regulatory cells [51,52]. This orchestration by MDSCs contributes significantly to immune evasion mechanisms within the tumor environment [51,52]. An increased infiltration of MDSCs in GC has been associated with poorer prognosis [53]. Dendritic cells are among the most potent antigen-presenting cells in the immune system [54]. In a cancer context, dendritic cells participate in a process called cross-priming, wherein they activate CD8^+^ T cells by presenting cancer antigens [55,56]. Subsequently, these CD8^+^ T cells undergo training and activation against the presented antigens, thereby initiating a cytotoxic immune response against tumor components [55,56]. This response constitutes a cornerstone of immunity against cancer antigens. Within the TME, various factors, such as IL-6, IL-10, Vascular Endothelial Growth Factor, and Transforming Growth Factor Beta, prevail [57,58]. These factors can negatively regulate the functions of dendritic cells, potentially leading to T-cell tolerance and immune escape rather than immunity [59,60,61].

*FN1* is a protein-coding gene responsible for encoding Fibronectin 1 [62]. Fibronectin, a high molecular weight glycoprotein [62], is found in the ECM. It plays crucial roles in various cellular functions, such as cell adhesion, growth, migration, and differentiation, contributing significantly to maintaining cell morphology [63]. Moreover, *FN1* displays chemotactic properties, attracting monocytes, and is involved in blood coagulation and wound healing processes [64]. Other bioinformatic in silico analyses have suggested a correlation between the overexpression of *FN1* and a poorer prognosis of patients with GC [65,66]. Our investigation substantiated these prior findings and demonstrated a link between FN1 overexpression and heightened infiltration of the TME and M2 macrophages. Concurrently, this gene was found to be associated with the reduced activity of dendritic cells.

*COL1A2* is responsible for encoding a chain integral to type I collagen, the predominant fibrillar collagen present in the majority of connective tissues [67]. *COL3A1* and COL5A1 encode the alpha-1 chains for type III and V collagen, respectively [67]. *COL1A2*, *COL3A1*, and *COL5A1* are highly prevalent structural proteins within the ECM. An earlier bioinformatic analysis has also revealed the correlation between *COL1A2*, *COL3A1*, *COL5A1*, *FN1*, and *CAF* infiltration in GC [66]. Furthermore, our study demonstrates that the expression of *COL1A2*, *COL3A1*, and *COL5A1* was positively correlated with M2 macrophages and negatively correlated with activated dendritic cells and CD8^+^ T-cell infiltration. Hence, we suggest that these genes may play an important role in maintaining and creating an immunosuppressive TME and subsequently promoting tumor progression.

*THBS2* encodes the protein Thrombospondin-2, a member of the matricellular calcium-binding glycoprotein family that interacts with growth factors, cell receptors, and the ECM [68]. Its functions include regulating cell proliferation, adhesion, and apoptosis. This gene has been extensively studied in relation to the prognosis and potential influence on the TME of gastric adenocarcinoma [69,70]. Zhang et al. have also constructed a nomogram centered around the expression of *THBS2* [70]. Our research validates that the increased expression of these genes correlates with a poorer survival rate linked to elevated levels of CAFs and M2 macrophage infiltration. A negative correlation with activated dendritic cell infiltration was also observed.

*BGN* is part of the small leucine-rich proteoglycans family and is responsible for encoding Biglycan, a protein that can be modified to form a glycoprotein [71]. BGN plays a role in cell proliferation, the migration of malignant cells, and reducing cell adhesion by interacting with proteins in both the intracellular and extracellular matrix [72,73]. Two in silico analyses have already demonstrated that high BGN expression is significantly associated with poor overall survival in GC patients [74,75]. Additionally, Chen et al. illustrated that BGN exhibited positive correlations with CD8^+^ T cells, macrophages, and dendritic cell infiltration in GC samples [74]. Zhang et al. also found, among others, a positive correlation between BGN expression and the infiltration of macrophages [75]. Our study partially confirms the former findings. In contrast to Chen et al.’s results [74], we observed a negative correlation with dendritic cells, potentially attributed to our focus on activated dendritic cells. Furthermore, akin to other genes, BGN displays a distinct positive correlation with CAFs. The aforementioned findings support the role of BGN in the occurrence and progression of GC, particularly through its influence on the TME.

## 5. Conclusions and Limitations

In this study, we identified immune-related genes and investigated the relation of the overexpression of these genes with cell infiltration in GC through bioinformatics analysis. The pathways involving focal adhesion, ECM–receptor interaction, and platelet activation appear to play a significant role in the pathogenesis and progression of GC, potentially serving as targets for pharmaceutical therapies. *FN1*, *COL3A1*, *COL5A1*, *BGN*, *THBS2*, and *COL1A2* offer promising biomarkers for the prognosis assessment of GC patients. Additionally, we observed a strong correlation between these genes and the composition of the TME. However, our study has several limitations that require careful consideration. Primarily, there is an urgent need for molecular experiments to validate our findings. Furthermore, our comparison was focused on paired GC tissues and their adjacent counterparts without accounting for crucial details such as histological type, GC grade, and the spatial relationship between adjacent and cancerous tissues. These factors could potentially influence the expression patterns of DEGs. Moreover, our identification of DEGs was confined to a single dataset, aiming to retain essential genes but potentially increasing the risk of false positives. Therefore, a larger sample size is essential to authenticate and substantiate the obtained results.

## Figures and Tables

**Table 1:** (a) Functional enrichment analyses GO: enriched biological processes. (b) Functional enrichment analyses GO: cellular components. (c) Functional enrichment analyses GO: molecular functions. (d) KEGG pathway enrichment analysis.

(a)								
ID		Term_Description	Fold_Enrichment	Occurrence	Support	Lowest_p	Highest_p	Up_Regulated
1	GO:0030199	collagen fibril organization	50.540767	10	0.065658240	5.736930e-08	5.736930e-08	BMP1, COL1A1, COL1A2, COL3A1, COL5A1, COL5A2, ADAMTS2
2	GO:0043588	skin development	41.876636	10	0.025253169	2.297293e-05	2.297293e-05	COL3A1, COL5A1, COL5A2
3	GO:0035987	endodermal cell differentiation	32.212797	10	0.030456853	4.686940e-05	4.686940e-05	COL8A1, COL12A1, FN1, INHBA
4	GO:0031175	neuron projection development	8.488507	10	0.010101268	1.162934e-03	1.162934e-03	APOE, MYOC, SH3GL2
5	GO:0001501	skeletal system development	22.334206	10	0.005050634	2.114611e-03	2.114611e-03	COL1A1, COL1A2, COL10A1, FBN1, HOXA13, TNFRSF11B, SOX4, TEAD4
6	GO:0003215	cardiac right ventricle morphogenesis	38.069669	10	0.012719036	2.225033e-03	5.559604e-03	GATA4, SOX4
7	GO:0016567	protein ubiquitination	2.448926	10	0.010101268	2.942256e-03	2.942256e-03	NFE2L2, KLHL25
8	GO:2001234	negative regulation of apoptotic signaling pathway	14.955941	10	0.005050634	3.680761e-03	3.680761e-03	GATA4
9	GO:0033700	phospholipid efflux	38.069669	10	0.005050634	3.707395e-03	3.707395e-03	APOC1, APOE
10	GO:0001568	blood vessel development	27.917757	10	0.025253169	4.246779e-03	4.246779e-03	COL1A1, COL1A2
(b)								
1	GO:0005788	endoplasmic reticulum lumen	13.232394	10	0.180195851	9.961557e-11	9.961557e-11	APOE, SERPINH1, COL1A1, COL1A2, COL3A1, COL5A1, COL5A2, COL6A3, COL8A1, COL10A1, COL12A1, FBN1, FN1, IGFBP4, SPP1, TIMP1, P4HA3
2	GO:0062023	collagen-containing extracellular matrix	18.207233	10	0.085860776	2.192636e-08	2.192636e-08	BGN, COL1A1, COL3A1, COL5A1, COL6A3, FN1, MYOC, SFRP2
3	GO:0031012	extracellular matrix	22.274806	10	0.095477387	1.306355e-05	1.306355e-05	APOE, COL6A3, ELN, FBN1, FN1
4	GO:0005793	endoplasmic reticulum–Golgi intermediate compartment	8.546252	10	0.050761421	1.484223e-05	7.398274e-05	SERPINH1, FN1
5	GO:0001527	microfibril	34.897196	10	0.015267274	5.961104e-04	9.932217e-04	FBN1, MFAP2
6	GO:0070062	extracellular exosome	3.579200	10	0.010101268	1.037442e-03	1.037442e-03	APOE, FN1
7	GO:0034361	very-low-density lipoprotein particle	29.911883	10	0.005050634	1.369197e-03	1.369197e-03	APOC1, APOE
8	GO:0071682	endocytic vesicle lumen	23.264798	10	0.010050251	1.381399e-03	6.434259e-03	APOE, SPARC
9	GO:0034364	high-density lipoprotein particle	27.917757	10	0.005050634	1.579702e-03	1.579702e-03	APOC1, APOE
10	GO:0031463	Cul3-RING ubiquitin ligase complex	6.158329	10	0.010101268	5.060305e-03	5.060305e-03	KLHL25
(c)								
1	GO:0048407	platelet-derived growth factor binding	76.139337	10	0.055556973	2.976699e-14	2.976699e-14	COL1A1, COL1A2, COL3A1, COL5A1
2	GO:0005201	extracellular matrix structural constituent	36.949973	10	0.106063311	6.443542e-06	6.443542e-06	COL3A1, ELN, FBN1, FN1, MUC5AC, NID2
3	GO:0002020	protease binding	12.316658	10	0.085860776	9.474422e-05	1.288232e-04	COL1A1, COL1A2, COL3A1, FAP, FN1
4	GO:0005178	integrin binding	15.592364	10	0.025253169	1.422341e-04	1.440477e-04	COL3A1, FAP, FBN1, FN1, SFRP2, SPP1, THY1
5	GO:0000976	transcription cis-regulatory region binding	3.172472	10	0.010101268	6.020621e-04	6.733792e-03	GATA4, NFE2L2, SOX4
6	GO:0048156	tau protein binding	16.106398	10	0.010101268	1.568042e-03	1.568042e-03	APOE
7	GO:0043395	heparan sulfate proteoglycan binding	17.448598	10	0.005050634	1.989792e-03	1.989792e-03	APOE
8	GO:0043394	proteoglycan binding	32.212797	10	0.025253169	2.351293e-03	2.351293e-03	COL5A1, FN1
9	GO:0030020	extracellular matrix structural constituent conferring tensile strength	41.876636	10	0.005050634	2.531860e-03	2.531860e-03	COL1A1, COL6A3
10	GO:0001968	fibronectin binding	19.941255	10	0.005050634	2.532614e-03	2.532614e-03	MYOC, SFRP2
(d)								
1	hsa04974	protein digestion and absorption	21.149816	10	0.100502513	5.555187e-11	5.555187e-11	ELN, COL1A1, COL1A2, COL3A1, COL5A1, COL5A2, COL6A3, COL8A1, COL10A1, COL12A1
2	hsa04512	ECM–receptor interaction	14.276126	10	0.061069098	1.036954e-06	1.036954e-06	COL1A1, COL1A2, COL6A3, THBS2, FN1, SPP1
3	hsa04510	focal adhesion	8.502870	10	0.035354437	2.685352e-05	2.685352e-05	COL1A1, COL1A2, COL6A3, THBS2, FN1, SPP1, PGF, TLN2
4	hsa04611	platelet activation	8.581278	10	0.010101268	1.964620e-04	1.964620e-04	TLN2, COL1A1, COL1A2, COL3A1, PLA2G4C
5	hsa05205	proteoglycans in cancer	3.094333	10	0.015151902	2.490706e-04	2.490706e-04	COL1A1, COL1A2, FN1
6	hsa04933	AGE-RAGE signaling pathway in diabetic complications	8.459926	10	0.055556973	5.189137e-04	5.189137e-04	FN1, COL1A1, COL1A2, COL3A1
7	hsa05146	amoebiasis	8.292403	10	0.055556973	5.512279e-04	5.512279e-04	COL1A1, COL1A2, FN1, COL3A1
8	hsa05415	diabetic cardiomyopathy	3.323543	10	0.010101268	7.352484e-04	7.352484e-04	COL1A1, COL1A2, COL3A1
9	hsa04926	relaxin signaling pathway	4.907418	10	0.010101268	1.126101e-03	1.126101e-03	COL1A1, COL1A2, COL3A1
10	hsa04310	Wnt signaling pathway	2.507583	10	0.005050634	1.259368e-03	1.259368e-03	SFRP2, SFRP4

## Data Availability

The datasets used in this study are all available in online public databases.

## Supplementary Materials

The following supporting information can be downloaded at: https://www.mdpi.com/article/10.3390/cancers16071280/s1, Figure S1: Pearson correlation between the infiltration level of the tumor-microenvironment component and the expression level of the genes; Table S1: Down-regulated genes and up-regulated genes in GSE19826; Table S2: Intersection of the top ten Hub DEGs.

## Abbreviations

BPs	Biological Processes
CAFs	Cancer-associated Fibroblasts
CCs	Cellular Components
DEGs	Differentially Expressed Genes
ECM	Extracellular Matrix
ECX	Epirubicin, Cisplatin, and Capecitabine
EMR	Endoscopic Mucosal Resection
ESD	Endoscopic Submucosal Dissection
FDA	Food and Drug Administration
FLOT	Fluorouracil, Oxaliplatin, Docetaxel, and Leucovorin
GC	Gastric Cancer/Gastric Carcinoma
GEO	Gene Expression Omnibus
GO	Gene Ontology
KEGG	Kyoto Encyclopedia of Genes and Genomes
MDSCs	Myeloid-derived Suppressor Cells
MFs	Molecular Functions
NFs	Normal Fibroblasts
PD-L1	Programmed Death-ligand 1
PPI	Protein–Protein Interaction
TAMs	Tumor-associated Macrophages
TIMER	Tumor Immune Evaluation Resource
TME	Tumor Microenvironment

## References

[1] Bray F., Ferlay J., Soerjomataram I., Siegel R.L., Torre L.A., Jemal A. (2018). Global cancer statistics 2018: GLOBOCAN estimates of incidence and mortality worldwide for 36 cancers in 185 countries. CA Cancer J. Clin..

[2] Carcas L.P. (2014). Gastric cancer review. J. Carcinog..

[3] Smyth E.C., Nilsson M., Grabsch H.I., van Grieken N.C., Lordick F. (2020). Gastric cancer. Lancet.

[4] Joshi S.S., Badgwell B.D. (2021). Current treatment and recent progress in gastric cancer. CA Cancer J. Clin..

[5] Rha S.Y., Oh D.-Y., Yañez P., Bai Y., Ryu M.-H., Lee J., Rivera F., Alves G.V., Garrido M., Shiu K.-K. (2023). Pembrolizumab plus chemotherapy versus placebo plus chemotherapy for HER2-negative advanced gastric cancer (KEYNOTE-859): A multicentre, randomised, double-blind, phase 3 trial. Lancet Oncol..

[6] Janjigian Y.Y., Shitara K., Moehler M., Garrido M., Salman P., Shen L., Wyrwicz L., Yamaguchi K., Skoczylas T., Bragagnoli A.C. (2021). First-line nivolumab plus chemotherapy versus chemotherapy alone for advanced gastric, gastro-oesophageal junction, and oesophageal adenocarcinoma (CheckMate 649): A randomised, open-label, phase 3 trial. Lancet.

[7] Anderson N.M., Simon M.C. (2020). The tumor microenvironment. Curr. Biol..

[8] Baghban R., Roshangar L., Jahanban-Esfahlan R., Seidi K., Ebrahimi-Kalan A., Jaymand M., Kolahian S., Javaheri T., Zare P. (2020). Tumor microenvironment complexity and therapeutic implications at a glance. Cell Commun. Signal..

[9] Li Y., Hu X., Lin R., Zhou G., Zhao L., Zhao D., Zhang Y., Li W., Zhang Y., Ma P. (2022). Single-cell landscape reveals active cell subtypes and their interaction in the tumor microenvironment of gastric cancer. Theranostics.

[10] Oliver G.R., Hart S.N., Klee E.W. (2015). Bioinformatics for clinical next generation sequencing. Clin. Chem..

[11] Zeng D., Li M., Zhou R., Zhang J., Sun H., Shi M., Bin J., Liao Y., Rao J., Liao W. (2019). Tumor microenvironment characterization in gastric cancer identifies prognostic and immunotherapeutically relevant gene signatures. Cancer Immunol. Res..

[12] Zeng D., Zhou R., Yu Y., Luo Y., Zhang J., Sun H., Bin J., Liao Y., Rao J., Zhang Y. (2018). Gene expression profiles for a prognostic immunoscore in gastric cancer. J. Br. Surg..

[13] Wei S., Lu J., Lou J., Shi C., Mo S., Shao Y., Ni J., Zhang W., Cheng X. (2020). Gastric cancer tumor microenvironment characterization reveals stromal-related gene signatures associated with macrophage infiltration. Front. Genet..

[14] Wang Q., Wen Y.-G., Li D.-P., Xia J., Zhou C.-Z., Yan D.-W., Tang H.-M., Peng Z.-H. (2012). Upregulated INHBA expression is associated with poor survival in gastric cancer. Med. Oncol..

[15] Li T., Fan J., Wang B., Traugh N., Chen Q., Liu J.S., Li B., Liu X.S. (2017). TIMER: A web server for comprehensive analysis of tumor-infiltrating immune cells. Cancer Res..

[16] Davis S., Meltzer P.S. (2007). GEOquery: A bridge between the Gene Expression Omnibus (GEO) and BioConductor. Bioinformatics.

[17] Ulgen E., Ozisik O., Sezerman O.U. (2019). pathfindR: An R package for comprehensive identification of enriched pathways in omics data through active subnetworks. Front. Genet..

[18] Kanehisa M., Goto S. (2000). KEGG: Kyoto encyclopedia of genes and genomes. Nucleic Acids Res..

[19] Szklarczyk D., Gable A.L., Nastou K.C., Lyon D., Kirsch R., Pyysalo S., Doncheva N.T., Legeay M., Fang T., Bork P. (2021). The STRING database in 2021: Customizable protein–protein networks, and functional characterization of user-uploaded gene/measurement sets. Nucleic Acids Res..

[20] Doncheva N.T., Morris J.H., Gorodkin J., Jensen L.J. (2018). Cytoscape StringApp: Network analysis and visualization of proteomics data. J. Proteome Res..

[21] Anaya J. (2016). OncoLnc: Linking TCGA survival data to mRNAs, miRNAs, and lncRNAs. PeerJ Comput. Sci..

[22] Li T., Fu J., Zeng Z., Cohen D., Li J., Chen Q., Li B., Liu X.S. (2020). TIMER2. 0 for analysis of tumor-infiltrating immune cells. Nucleic Acids Res..

[23] Guo W., Giancotti F.G. (2004). Integrin signalling during tumour progression. Nat. Rev. Mol. Cell Biol..

[24] Petit V., Thiery J.-P. (2000). Focal adhesions: Structure and dynamics. Biol. Cell.

[25] Peng K., Li S., Li Q., Zhang C., Yuan Y., Liu M., Zhang L., Wang Y., Yu S., Zhang H. (2022). Positive Phospho-Focal Adhesion Kinase in Gastric Cancer Associates With Poor Prognosis After Curative Resection. Front. Oncol..

[26] Gay L.J., Felding-Habermann B. (2011). Contribution of platelets to tumour metastasis. Nat. Rev. Cancer.

[27] Palacios-Acedo A.L., Mège D., Crescence L., Dignat-George F., Dubois C., Panicot-Dubois L. (2019). Platelets, thrombo-inflammation, and cancer: Collaborating with the enemy. Front. Immunol..

[28] Ferroni P., Martini F., Portarena I., Grenga I., Riondino S., La Farina F., Laudisi A., Roselli M., Guadagni F. (2011). An activated protein C-dependent thrombin generation assay predicts chemotherapy-associated venous thromboembolism in cancer patients. Thromb. Haemost..

[29] Meikle C.K., Kelly C.A., Garg P., Wuescher L.M., Ali R.A., Worth R.G. (2017). Cancer and thrombosis: The platelet perspective. Front. Cell Dev. Biol..

[30] Asai K., Hirabayashi T., Houjou T., Uozumi N., Taguchi R., Shimizu T. (2003). Human group IVC phospholipase A2 (cPLA2γ): Roles in the membrane remodeling and activation induced by oxidative stress. J. Biol. Chem..

[31] Albini A., Magnani E., Noonan D. (2010). The tumor microenvironment: Biology of a complex cellular and tissue society. Q. J. Nucl. Med. Mol. Imaging.

[32] Sun H., Wang X., Wang X., Xu M., Sheng W. (2022). The role of cancer-associated fibroblasts in tumorigenesis of gastric cancer. Cell Death Dis..

[33] Orimo A., Gupta P.B., Sgroi D.C., Arenzana-Seisdedos F., Delaunay T., Naeem R., Carey V.J., Richardson A.L., Weinberg R.A. (2005). Stromal fibroblasts present in invasive human breast carcinomas promote tumor growth and angiogenesis through elevated SDF-1/CXCL12 secretion. Cell.

[34] Cui X., Shan T., Qiao L. (2022). Collagen type IV alpha 1 (COL4A1) silence hampers the invasion, migration and epithelial–mesenchymal transition (EMT) of gastric cancer cells through blocking Hedgehog signaling pathway. Bioengineered.

[35] Izumi D., Ishimoto T., Miyake K., Sugihara H., Eto K., Sawayama H., Yasuda T., Kiyozumi Y., Kaida T., Kurashige J. (2016). CXCL12/CXCR4 activation by cancer-associated fibroblasts promotes integrin β1 clustering and invasiveness in gastric cancer. Int. J. Cancer.

[36] Zhang H., Deng T., Liu R., Ning T., Yang H., Liu D., Zhang Q., Lin D., Ge S., Bai M. (2020). CAF secreted miR-522 suppresses ferroptosis and promotes acquired chemo-resistance in gastric cancer. Mol. Cancer.

[37] Liu X., Yao L., Qu J., Liu L., Lu N., Wang J., Zhang J. (2021). Cancer-associated fibroblast infiltration in gastric cancer: The discrepancy in subtypes pathways and immunosuppression. J. Transl. Med..

[38] Trujillo J.A., Sweis R.F., Bao R., Luke J.J. (2018). T cell–inflamed versus non-T cell–inflamed tumors: A conceptual framework for cancer immunotherapy drug development and combination therapy selection. Cancer Immunol. Res..

[39] Galon J., Bruni D. (2019). Approaches to treat immune hot, altered and cold tumours with combination immunotherapies. Nat. Rev. Drug Discov..

[40] Nolz J.C. (2015). Molecular mechanisms of CD8+ T cell trafficking and localization. Cell. Mol. Life Sci..

[41] Slaney C.Y., Kershaw M.H., Darcy P.K. (2014). Trafficking of T cells into tumors. Cancer Res..

[42] Li F., Li C., Cai X., Xie Z., Zhou L., Cheng B., Zhong R., Xiong S., Li J., Chen Z. (2021). The association between CD^8+^ tumor-infiltrating lymphocytes and the clinical outcome of cancer immunotherapy: A systematic review and meta-analysis. EClinicalMedicine.

[43] Cui C., Zhang D., Sun K., Zhu Y., Xu J., Kang Y., Zhang G., Cai Y., Mao S., Long R. (2022). Propofol maintains Th17/Treg cell balance in elderly patients undergoing lung cancer surgery through GABAA receptor. BMC Immunol..

[44] Ginhoux F., Jung S. (2014). Monocytes and macrophages: Developmental pathways and tissue homeostasis. Nat. Rev. Immunol..

[45] Chanmee T., Ontong P., Konno K., Itano N. (2014). Tumor-associated macrophages as major players in the tumor microenvironment. Cancers.

[46] Lee K.Y. (2019). M1 and M2 polarization of macrophages: A mini-review. Med. Biol. Sci. Eng..

[47] Wang N., Liang H., Zen K. (2014). Molecular mechanisms that influence the macrophage M1–M2 polarization balance. Front. Immunol..

[48] Gao J., Liang Y., Wang L. (2022). Shaping polarization of tumor-associated macrophages in cancer immunotherapy. Front. Immunol..

[49] Schulz C., Perdiguero E.G., Chorro L., Szabo-Rogers H., Cagnard N., Kierdorf K., Prinz M., Wu B., Jacobsen S.E.W., Pollard J.W. (2012). A lineage of myeloid cells independent of Myb and hematopoietic stem cells. Science.

[50] Gabrilovich D.I., Ostrand-Rosenberg S., Bronte V. (2012). Coordinated regulation of myeloid cells by tumours. Nat. Rev. Immunol..

[51] Solito S., Bronte V., Mandruzzato S. (2011). Antigen specificity of immune suppression by myeloid-derived suppressor cells. J. Leukoc. Biol..

[52] Yang Z., Guo J., Weng L., Tang W., Jin S., Ma W. (2020). Myeloid-derived suppressor cells—New and exciting players in lung cancer. J. Hematol. Oncol..

[53] Gabitass R.F., Annels N.E., Stocken D.D., Pandha H.A., Middleton G.W. (2011). Elevated myeloid-derived suppressor cells in pancreatic, esophageal and gastric cancer are an independent prognostic factor and are associated with significant elevation of the Th2 cytokine interleukin-13. Cancer Immunol. Immunother..

[54] Banchereau J., Steinman R.M. (1998). Dendritic cells and the control of immunity. Nature.

[55] Kurts C., Robinson B.W., Knolle P.A. (2010). Cross-priming in health and disease. Nat. Rev. Immunol..

[56] Melief C.J. (2008). Cancer immunotherapy by dendritic cells. Immunity.

[57] Gottfried E., Kreutz M., Mackensen A. (2008). Tumor-induced modulation of dendritic cell function. Cytokine Growth Factor Rev..

[58] Zong J., Keskinov A.A., Shurin G.V., Shurin M.R. (2016). Tumor-derived factors modulating dendritic cell function. Cancer Immunol. Immunother..

[59] Bandola-Simon J., Roche P.A. (2019). Dysfunction of antigen processing and presentation by dendritic cells in cancer. Mol. Immunol..

[60] Chrisikos T.T., Zhou Y., Slone N., Babcock R., Watowich S.S., Li H.S. (2019). Molecular regulation of dendritic cell development and function in homeostasis, inflammation, and cancer. Mol. Immunol..

[61] Gupta Y.H., Khanom A., Acton S.E. (2022). Control of dendritic cell function within the tumour microenvironment. Front. Immunol..

[62] Mitrović S., Mitrović D., Todorović V. (1995). Fibronectin—A multifunctional glycoprotein. Srp. Arh. Za Celok. Lek..

[63] Pankov R., Yamada K.M. (2002). Fibronectin at a glance. J. Cell Sci..

[64] Grinnell F., Billingham R.E., Burgess L. (1981). Distribution of fibronectin during wound healing in vivo. J. Investig. Dermatol..

[65] Liu J., Ma L., Chen Z., Song Y., Gu T., Liu X., Zhao H., Yao N. (2020). Identification of critical genes in gastric cancer to predict prognosis using bioinformatics analysis methods. Ann. Transl. Med..

[66] Ucaryilmaz Metin C., Ozcan G. (2022). Comprehensive bioinformatic analysis reveals a cancer-associated fibroblast gene signature as a poor prognostic factor and potential therapeutic target in gastric cancer. BMC Cancer.

[67] Gelse K., Pöschl E., Aigner T. (2003). Collagens—Structure, function, and biosynthesis. Adv. Drug Deliv. Rev..

[68] Adams J.C., Lawler J. (2004). The thrombospondins. Int. J. Biochem. Cell Biol..

[69] Liao X., Wang W., Yu B., Tan S. (2022). Thrombospondin-2 acts as a bridge between tumor extracellular matrix and immune infiltration in pancreatic and stomach adenocarcinomas: An integrative pan-cancer analysis. Cancer Cell Int..

[70] Zhang S., Yang H., Xiang X., Liu L., Huang H., Tang G. (2022). THBS2 is closely related to the poor prognosis and immune cell infiltration of gastric cancer. Front. Genet..

[71] Chen D., Qin Y., Dai M., Li L., Liu H., Zhou Y., Qiu C., Chen Y., Jiang Y. (2020). BGN and COL11A1 regulatory network analysis in colorectal cancer (CRC) reveals that BGN influences CRC cell biological functions and interacts with miR-6828-5p. Cancer Manag. Res..

[72] Cooper J., Giancotti F.G. (2019). Integrin signaling in cancer: Mechanotransduction, stemness, epithelial plasticity, and therapeutic resistance. Cancer Cell.

[73] Moreno-Layseca P., Icha J., Hamidi H., Ivaska J. (2019). Integrin trafficking in cells and tissues. Nat. Cell Biol..

[74] Chen W., Yang Z. (2021). Identification of differentially expressed genes reveals BGN predicting overall survival and tumor immune infiltration of gastric cancer. Comput. Math. Methods Med..

[75] Zhang S., Yang H., Xiang X., Liu L., Huang H., Tang G. (2022). BGN may be a potential prognostic biomarker and associated with immune cell enrichment of gastric cancer. Front. Genet..

