# Supplementary material for: Identification of Key Genes Associated with Tumor Microenvironment Infiltration and Survival in Gastric Adenocarcinoma via Bioinformatics Analysis
Source: Cancers (Basel). 2024 Mar 26;16(7):1280. doi: 10.3390/cancers16071280 (PMC11010876; doi:10.3390/cancers16071280)
Supplement: Supplementary file 1 [file cancers-16-01280-s001.zip › Gastric_Cancer_Supp. Table S2.pdf]

Supp. Table S2. Intersection of the top ten Hub DEGs.

**Top 10 ranked by BottleNeck method**		
**Rank**	Name	Score
**1**	FN1	27.0
**2**	SPP1	13.0
**3**	APOE	8.0
**4**	COL1A2	5.0
**4**	VTN	5.0
**6**	COL1A1	3.0
**6**	LIF	3.0
**6**	GATA4	3.0
**6**	BGN	3.0
**6**	NANOG	3.0
**Top 10 ranked by Degree method**		
**Rank**	Name	Score
**1**	FN1	39.0
**2**	COL1A1	29.0
**3**	BGN	25.0
**4**	COL1A2	24.0
**4**	SPP1	24.0
**4**	COL3A1	24.0

**7**	THBS2	21.0
**8**	COL5A1	20.0
**9**	APOE	19.0
**9**	TIMP1	19.0
**Top 10 ranked by MCC method**		
**Rank**	Name	Score
**1**	COL1A1	3.7164295E7
**2**	COL1A2	3.7148928E7
**3**	COL3A1	3.714572E7
**4**	COL5A1	3.7144848E7
**5**	COL5A2	3.6973566E7
**6**	SERPINH1	3.673152E7
**7**	COL10A1	3.665664E7
**8**	FN1	2.2127592E7
**9**	COL6A3	2.193948E7
**10**	COL8A1	2.193408E7
**Top 10 ranked by EPC method**		
**Rank**	Name	Score
**1**	FN1	23.122999999999993
**2**	COL1A1	22.640999999999994
**3**	BGN	22.335999999999956

**4**	COL1A2	22.20499999999994
**5**	COL3A1	21.976999999999965
**6**	COL5A1	21.200999999999965
**7**	SPP1	20.432999999999957
**8**	THBS2	20.39299999999997
**9**	COL6A3	20.373999999999977
**10**	SPARC	20.179999999999986
**Top 10 ranked by MNC method**		
**Rank**	Name	Score
**1**	FN1	39.0
**2**	COL1A1	28.0
**3**	BGN	25.0
**4**	COL1A2	24.0
**4**	SPP1	24.0
**4**	COL3A1	24.0
**7**	COL5A1	20.0
**7**	THBS2	20.0
**9**	TIMP1	19.0
**10**	APOE	18.0
