# Supplementary material for: Identification of Key Genes Associated with Tumor Microenvironment Infiltration and Survival in Gastric Adenocarcinoma via Bioinformatics Analysis
Source: Cancers (Basel). 2024 Mar 26;16(7):1280. doi: 10.3390/cancers16071280 (PMC11010876; doi:10.3390/cancers16071280)
Supplement: Supplementary file 1 [file cancers-16-01280-s001.zip › Gastric_Cancer_Suppl. Table S1.pdf]

Table S1: Down-regulated genes and up-regulated genes in GSE19826

ID	Gene.symbol	Gene.title	log2(fold change)	-log10(Pvalue)
**1552448_a_at**	FAM167A-AS1	FAM167A antisense RNA 1	-3.328	4.524
**1552466_x_at**	LINC00161	long intergenic non-protein coding RNA 161	-2.519	3.852
**1552755_at**	C9orf66	chromosome 9 open reading frame 66	-2.249	3.83
**1553074_at**	ASB11	ankyrin repeat and SOCS box containing 11	-2.54	4.245
**1553131_a_at**	GATA4	GATA binding protein 4	-2.067	4.208
**1553174_at**	JPH2	junctophilin 2	-2.201	3.608
**1554018_at**	GPNMB	glycoprotein nmb	1.716	3.889
**1554810_at**	PLA2G4C	phospholipase A2 group IVC	1.924	3.561
**1555018_at**	OR2C3	olfactory receptor family 2 subfamily C member 3	-2.95	5.61
**1556368_at**	PHKG2	phosphorylase kinase catalytic subunit gamma 2	-2.03	3.59
**1556903_at**	LOC101928335	uncharacterized LOC101928335	-2.709	3.768
**1557181_s_at**	C11orf87	chromosome 11 open reading frame 87	-2.203	4.797
**1560019_at**	DLGAP1-AS2	DLGAP1 antisense RNA 2	2.416	4.047
**1560725_at**	IL12A-AS1	IL12A antisense RNA 1	-2.056	3.645
**1564233_at**	FLJ33534	uncharacterized LOC285150	-1.748	4.012

**1564315_at**	C8orf49	chromosome 8 open reading frame 49	-1.662	5.026
**1564333_a_at**	PSAPL1	prosaposin-like 1 (gene/pseudogene)	-2.807	4.372
**1565666_s_at**	MUC6	mucin 6, oligomeric mucus/gel-forming	-4.305	3.871
**1567013_at**	NFE2L2	nuclear factor, erythroid 2 like 2	-1.537	4.133
**1568574_x_at**	SPP1	secreted phosphoprotein 1	2.908	3.651
**1569150_x_at**	PDLIM7	PDZ and LIM domain 7	2.396	3.614
**1569688_at**	FMO5	flavin containing monooxygenase 5	-2.056	3.896
**1569900_at**	LOC101927533///LOC440867	uncharacterized LOC101927533///uncharacterized LOC440867	-1.672	4.301
**1570153_at**	CCDC169	coiled-coil domain containing 169	-2.378	4.124
**1570627_at**	TCEB3	transcription elongation factor B subunit 3	-2.077	3.73
**200665_s_at**	SPARC	secreted protein acidic and cysteine rich	1.647	5.605
**200938_s_at**	RERE	arginine-glutamic acid dipeptide repeats	-2.09	4.808
**201261_x_at**	BGN	biglycan	2.255	4.501
**201262_s_at**	BGN	biglycan	1.797	4.109

**201402_at**	GRK2	G protein-coupled receptor kinase 2	-2.015	3.746
**201418_s_at**	SOX4	SRY-box 4	1.549	4.34
**201438_at**	COL6A3	collagen type VI alpha 3 chain	1.874	6.335
**201508_at**	IGFBP4	insulin like growth factor binding protein 4	1.575	4.028
**201534_s_at**	UBL3	ubiquitin like 3	-1.574	4.487
**201666_at**	TIMP1	TIMP metallopeptidase inhibitor 1	1.815	4.137
**202310_s_at**	COL1A1	collagen type I alpha 1 chain	2.241	6.003
**202311_s_at**	COL1A1	collagen type I alpha 1 chain	2.568	5.434
**202404_s_at**	COL1A2	collagen type I alpha 2 chain	2.58	6.65
**202766_s_at**	FBN1	fibrillin 1	1.576	4.065
**203083_at**	THBS2	thrombospondin 2	2.732	4.156
**203381_s_at**	APOE	apolipoprotein E	1.839	4.26
**203417_at**	MFAP2	microfibrillar associated protein 2	2.278	5.867
**204051_s_at**	SFRP4	secreted frizzled related protein 4	2.471	3.563
**204073_s_at**	MYRF	myelin regulatory factor	-1.633	3.739
**204114_at**	NID2	nidogen 2	1.688	4.61
**204281_at**	TEAD4	TEA domain transcription factor 4	1.964	3.886
**204779_s_at**	HOXB7	homeobox B7	1.836	4.127

**204933_s_at**	TNFRSF11B	TNF receptor superfamily member 11b	2.862	3.573
**205470_s_at**	KLK11	kallikrein related peptidase 11	-2.783	3.705
**205574_x_at**	BMP1	bone morphogenetic protein 1	2.178	3.879
**205580_s_at**	HRH1	histamine receptor H1	2.051	4.665
**205751_at**	SH3GL2	SH3 domain containing GRB2 like 2, endophilin A1	-3.92	3.779
**205876_at**	LIFR	leukemia inhibitory factor receptor alpha	-3.118	4.416
**205941_s_at**	COL10A1	collagen type X alpha 1 chain	3.801	4.317
**205942_s_at**	ACSM3	acyl-CoA synthetase medium-chain family member 3	-2.008	3.882
**206469_x_at**	AKR7A3	aldo-keto reductase family 7 member A3	-2.344	4.124
**207331_at**	CENPF	centromere protein F	2.073	3.686
**207714_s_at**	SERPINH1	serpin family H member 1	2.786	5.824
**208321_s_at**	CABP1	calcium binding protein 1	-1.924	3.899
**208432_s_at**	CACNA1E	calcium voltage-gated channel subunit alpha1 E	-1.714	3.88
**208850_s_at**	THY1	Thy-1 cell surface antigen	1.892	4.342
**208851_s_at**	THY1	Thy-1 cell surface antigen	1.718	3.818
**209652_s_at**	PGF	placental growth factor	1.892	4.15
**209703_x_at**	METTL7A	methyltransferase like 7A	-1.605	3.802

**209955_s_at**	FAP	fibroblast activation protein alpha	3.768	5.333
**210155_at**	MYOC	myocilin	-2.488	3.943
**210307_s_at**	KLHL25	kelch like family member 25	1.923	5.261
**210495_x_at**	FN1	fibronectin 1	1.713	4.799
**210511_s_at**	INHBA	inhibin beta A subunit	4.807	5.391
**210619_s_at**	HYAL1	hyaluronoglucosaminidase 1	-2.234	4.259
**211062_s_at**	GPR78///CPZ	G protein-coupled receptor 78///carboxypeptidase Z	3.11	4.66
**211161_s_at**	COL3A1	collagen type III alpha 1 chain	1.622	4.904
**211689_s_at**	TMPRSS2	transmembrane protease, serine 2	-1.852	3.797
**211709_s_at**	CLEC11A	C-type lectin domain family 11 member A	2.115	3.854
**211719_x_at**	FN1	fibronectin 1	1.718	5.017
**212353_at**	SULF1	sulfatase 1	2.246	4.215
**212354_at**	SULF1	sulfatase 1	2.501	4.531
**212464_s_at**	FN1	fibronectin 1	1.723	4.591
**212488_at**	COL5A1	collagen type V alpha 1 chain	1.77	6.42
**213147_at**	HOXA10	homeobox A10	2.652	3.582
**213150_at**	HOXA10	homeobox A10	3.871	4.703
**213553_x_at**	APOC1	apolipoprotein C1	1.822	4.411
**213869_x_at**	THY1	Thy-1 cell surface antigen	1.774	4.092
**213905_x_at**	BGN	biglycan	2.025	4.764

**214502_at**	HIST1H2BJ	histone cluster 1, H2bj	1.994	4.348
**215077_at**	COL3A1	collagen type III alpha 1 chain	2.074	4.077
**215100_at**	ADTRP	androgen dependent TFPI regulating protein	-1.804	3.627
**215140_at**			-1.705	3.645
**215805_at**			-1.981	3.653
**215833_s_at**	SPPL2B	signal peptide peptidase like 2B	1.687	3.569
**216381_x_at**	AKR7A3	aldo-keto reductase family 7 member A3	-2.185	4.158
**216442_x_at**	FN1	fibronectin 1	1.801	5.014
**216552_x_at**	KIR2DS4	killer cell immunoglobulin like receptor, two Ig domains and short cytoplasmic tail 4	-2.342	3.95
**216994_s_at**	RUNX2	runt related transcription factor 2	-1.867	3.827
**217034_at**	LOC101928457	uncharacterized LOC101928457	-2.024	4.38
**217187_at**	MUC5AC	mucin 5AC, oligomeric mucus/gel-forming	-3.091	6.206
**220304_s_at**	CNGB3	cyclic nucleotide gated channel beta 3	-2.264	4.115
**220405_at**	SNTG1	syntrophin gamma 1	-2.232	3.839
**220636_at**	DNAI2	dynein axonemal intermediate chain 2	-2.119	4.088
**220819_at**	FRMD1	FERM domain containing 1	-2.065	4.003
**221320_at**	BCL2L10	BCL2 like 10	-1.729	3.737

**221729_at**	COL5A2	collagen type V alpha 2 chain	1.501	5.225
**222080_s_at**	SIRT5	sirtuin 5	-1.6	3.642
**223121_s_at**	SFRP2	secreted frizzled related protein 2	2.487	3.896
**224134_at**	MGC10814	uncharacterized protein MGC10814	-1.701	4.318
**225571_at**	LIFR	leukemia inhibitory factor receptor alpha	-2.5	4.96
**226237_at**	COL8A1	collagen type VIII alpha 1 chain	2.933	5.311
**226306_at**	C6orf1	chromosome 6 open reading frame 1	1.891	4.353
**226311_at**	ADAMTS2	ADAM metallopeptidase with thrombospondin type 1 motif 2	2.851	4.772
**226597_at**	REEP6	receptor accessory protein 6	2.637	4.454
**226695_at**	PRRX1	paired related homeobox 1	1.87	4.999
**226828_s_at**	HEYL	hes related family bHLH transcription factor with YRPW motif-like	1.543	4.049
**226997_at**	ADAMTS12	ADAM metallopeptidase with thrombospondin type 1 motif 12	2.363	3.964
**227140_at**	INHBA	inhibin beta A subunit	4.163	5.804
**227614_at**	HKDC1	hexokinase domain containing 1	2.573	3.86
**227771_at**	LIFR	leukemia inhibitory factor receptor alpha	-2.088	5.104
**228231_at**	MIR6836///SNX8	microRNA 6836///sorting nexin 8	2.202	3.864

**228703_at**	P4HA3	prolyl 4-hydroxylase subunit alpha 3	2.549	4.864
**229094_at**	ATP6V0E2-AS1	ATP6V0E2 antisense RNA 1	-2.181	3.862
**229176_at**	ANKH	ANKH inorganic pyrophosphate transport regulator	1.845	4.507
**229218_at**	COL1A2	collagen type I alpha 2 chain	2.001	5.036
**229254_at**	MFSD4A	major facilitator superfamily domain containing 4A	-3.914	3.574
**229661_at**	SALL4	spalt like transcription factor 4	3.063	4.268
**230271_at**	ONECUT2	one cut homeobox 2	2.665	4.578
**230371_at**	HPS6	HPS6, biogenesis of lysosomal organelles complex 2 subunit 3	-1.903	4.064
**231014_at**	TRIM50	tripartite motif containing 50	-2.131	3.846
**231646_at**	DPCR1	diffuse panbronchiolitis critical region 1	-4.871	4.182
**231766_s_at**	COL12A1	collagen type XII alpha 1 chain	1.664	4.493
**231786_at**	HOXA13	homeobox A13	4.338	5.378
**232478_at**	MIR181A2HG	MIR181A2 host gene	1.981	3.561
**232625_at**	TLN2	talin 2	-1.638	4.288
**232736_s_at**	POM121L10P///POM121L8P///POM121L9P	POM121 transmembrane nucleoporin like 10, pseudogene///POM121 transmembrane nucleoporin like 8, pseudogene///POM121	1.886	3.565

		transmembrane nucleoporin like 9, pseudogene		
**233184_at**	EPHA6	EPH receptor A6	-2.276	3.581
**233446_at**	ONECUT2	one cut homeobox 2	3.421	5.265
**233634_at**	MARVELD3	MARVEL domain containing 3	2.074	3.936
**233643_at**	FAM90A2P///FAM90A25P///FAM86B3P///FAM90A1	family with sequence similarity 90, member A2 pseudogene///family with sequence similarity 90, member A7 pseudogene///family with sequence similarity 86, member A pseudogene///family with sequence similarity 90 member A1	-2.292	5.716
**233978_at**	PTPRE	protein tyrosine phosphatase, receptor type E	-1.828	4.07
**234370_at**	VSIG1	V-set and immunoglobulin domain containing 1	-3.523	3.675
**234691_at**	KRTAP2-1	keratin associated protein 2-1	-1.889	3.574
**234999_at**	C19orf47	chromosome 19 open reading frame 47	-1.502	3.916
**235050_at**	SLC2A12	solute carrier family 2 member 12	-1.769	3.567
**235638_at**	RASSF6	Ras association domain family member 6	-2.11	4.086
**236098_at**	SMIM6	small integral membrane protein 6	-1.547	3.977

**237805_at**	LOC729296	uncharacterized LOC729296	-2.088	3.636
**238019_at**	PLIN5	perilipin 5	-1.774	4.01
**238205_at**	DCAF12L1	DDB1 and CUL4 associated factor 12 like 1	-2.963	3.613
**238481_at**	MGP	matrix Gla protein	2.165	4.617
**238623_at**			1.984	3.997
**238663_x_at**	GRIA4	glutamate ionotropic receptor AMPA type subunit 4	-3.735	4.533
**238862_at**	MFSD4A	major facilitator superfamily domain containing 4A	-3.684	4.111
**241137_at**	DPCR1	diffuse panbronchiolitis critical region 1	-4.212	3.837
**241502_x_at**			-2.07	3.623
**242222_at**	LINC01123///LINC01106	long intergenic non-protein coding RNA 1123///long intergenic non-protein coding RNA 1106	1.695	3.983
**242913_at**	CLIC6	chloride intracellular channel 6	-2.425	3.747
**242998_at**	RDH12	retinol dehydrogenase 12 (all-trans/9-cis/11-cis)	-2.689	3.569
**243749_s_at**	MINCR	MYC-induced long noncoding RNA	-1.726	4.175
**243764_at**	VSIG1	V-set and immunoglobulin domain containing 1	-3.612	3.583
