# Supplementary material for: Identification of Key Genes Associated with Tumor Microenvironment Infiltration and Survival in Gastric Adenocarcinoma via Bioinformatics Analysis
Source: Cancers (Basel). 2024 Mar 26;16(7):1280. doi: 10.3390/cancers16071280 (PMC11010876; doi:10.3390/cancers16071280)
Supplement: Supplementary file 1 [file cancers-16-01280-s001.zip › Gastric_Cancer_Supplementary Figure S1.pdf]

Figure S1. Pearson correlation between the infiltration level of the tumor-microenvironment component and the expression level of the genes.

a) BGN

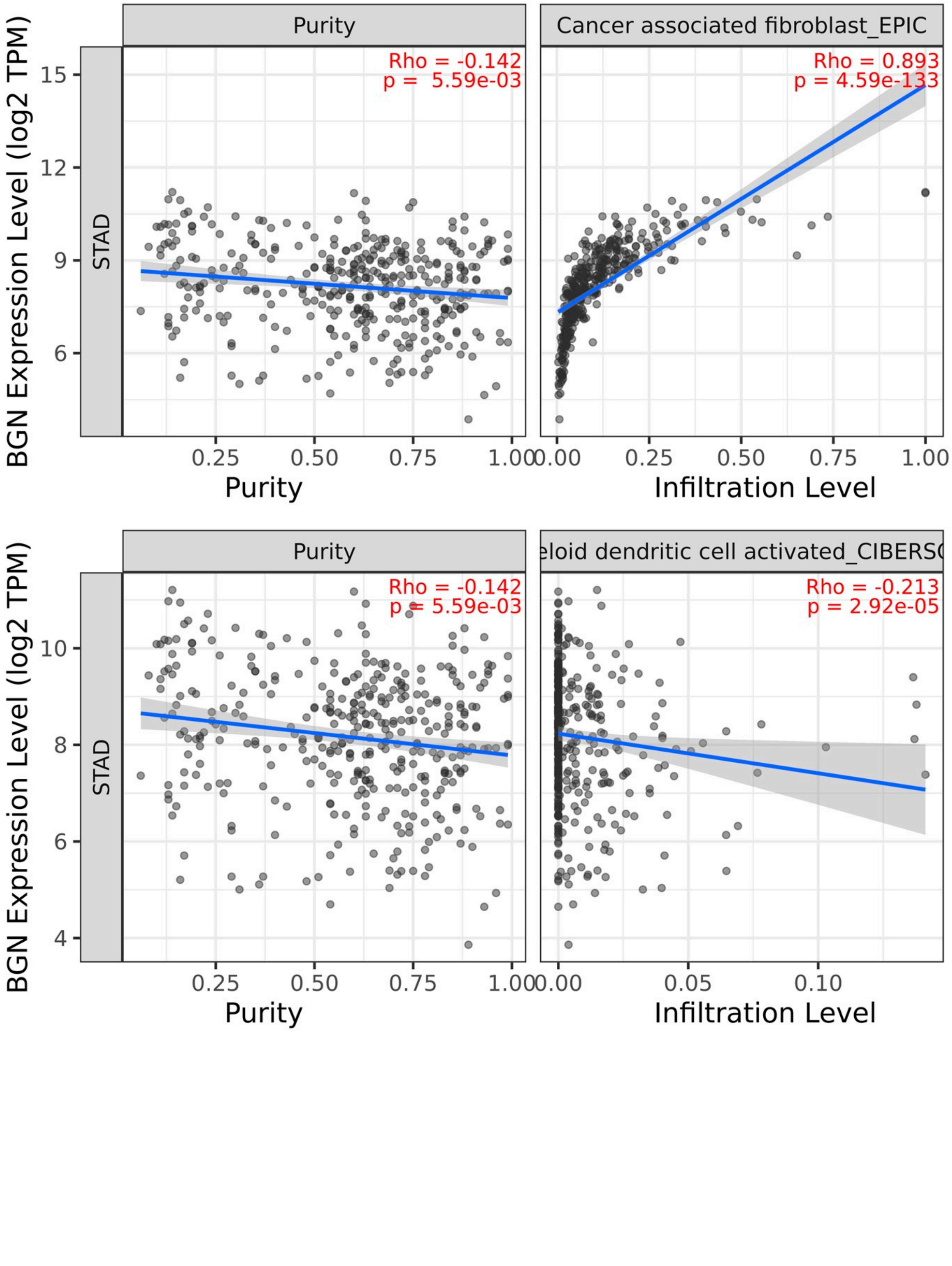

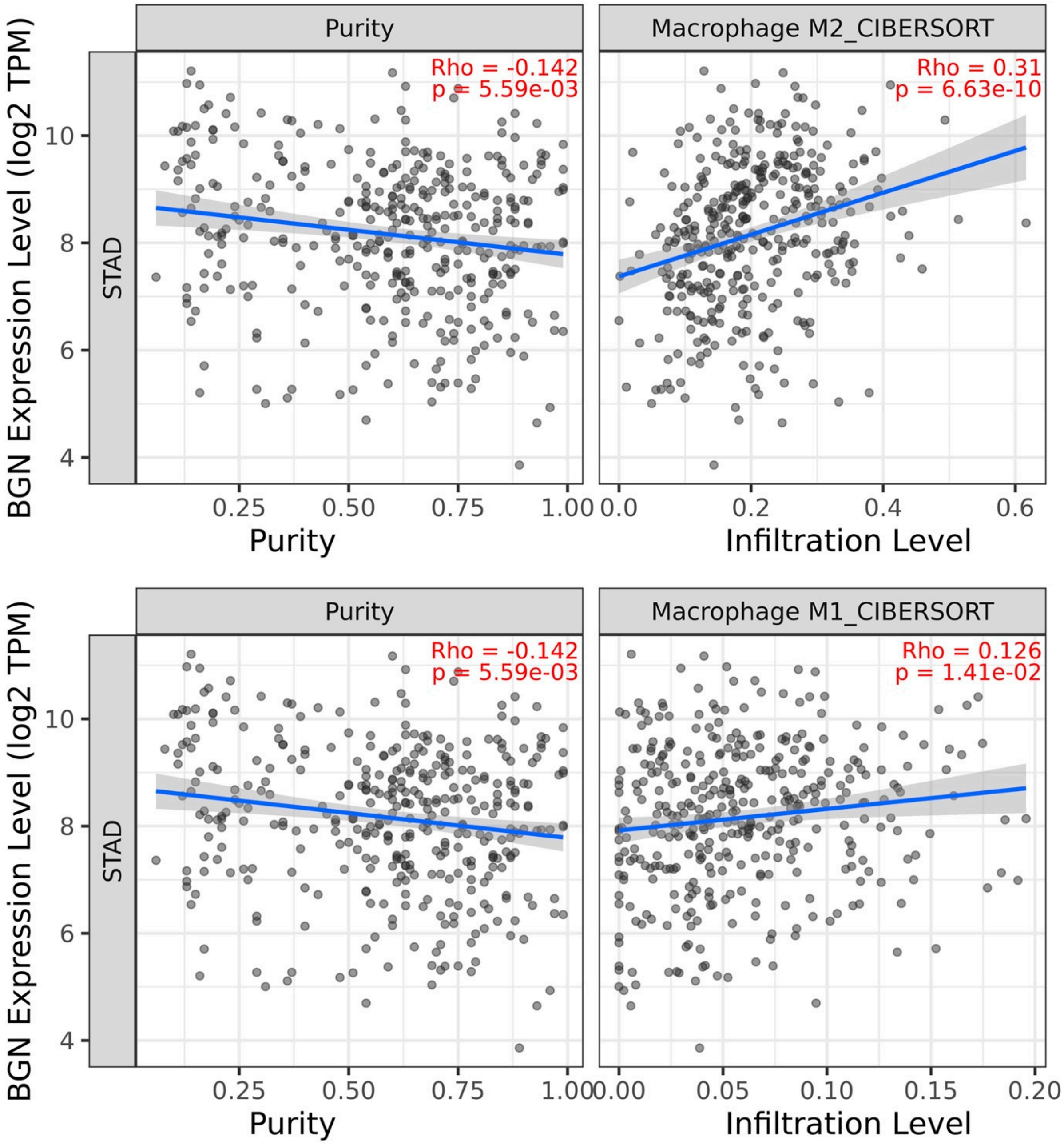
Purity
Macrophage M2_CIBERSORT
Rho = -0.142
p = 5.59e-03
Rho = 0.31
p = 6.63e-10
STAD
BGN Expression Level (log2 TPM)
Purity
Infiltration Level
Purity
Macrophage M1_CIBERSORT
Rho = -0.142
p = 5.59e-03
Rho = 0.126
p = 1.41e-02
STAD
BGN Expression Level (log2 TPM)
Purity
Infiltration Level

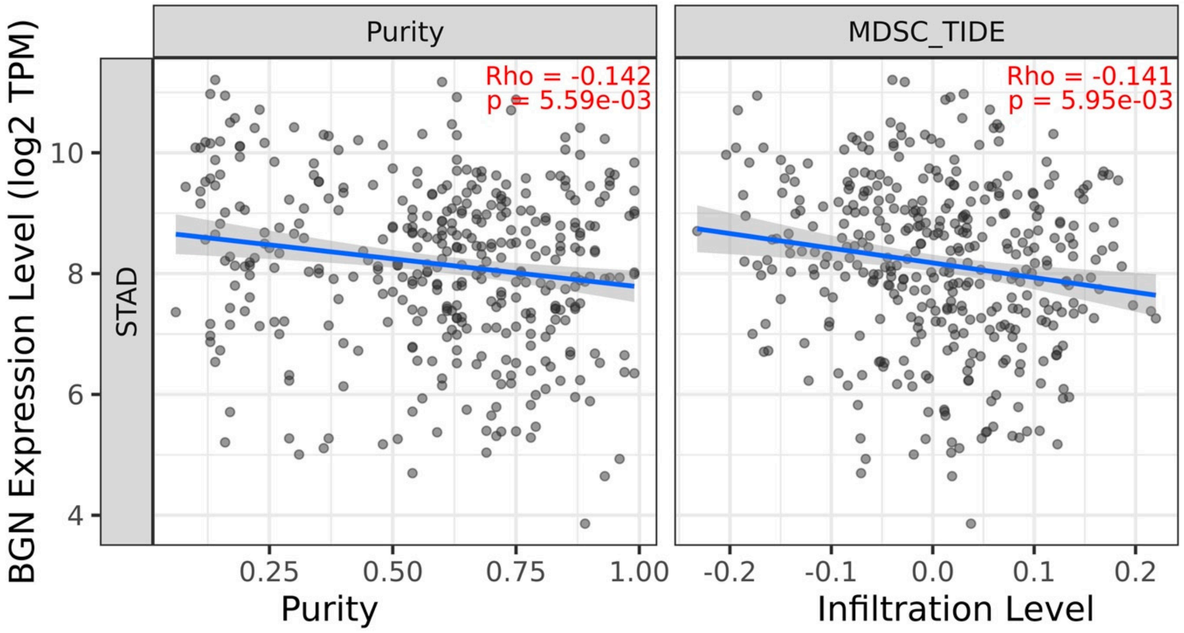
Purity
MDSC_TIDE
Rho = -0.142
p = 5.59e-03
Rho = -0.141
p = 5.95e-03
BGN Expression Level (log2 TPM)
STAD
10
8
6
4
0.25
0.50
0.75
1.00
-0.2
-0.1
0.0
0.1
0.2
Purity
Infiltration Level

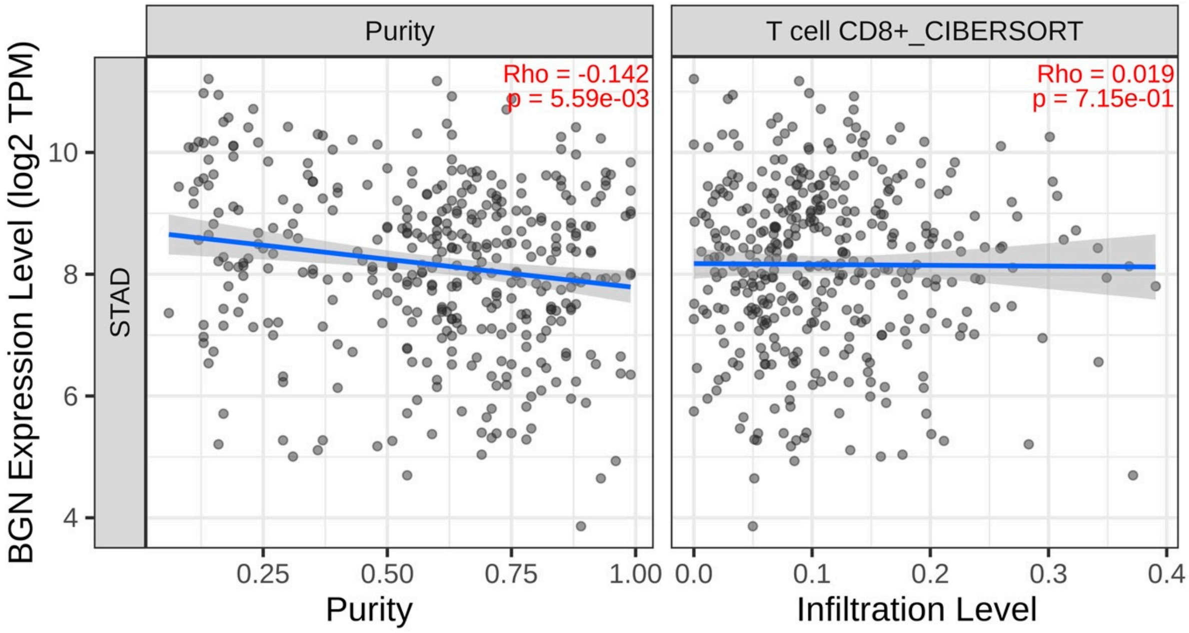
Purity
T cell CD8+_CIBERSORT
Rho = -0.142
p = 5.59e-03
Rho = 0.019
p = 7.15e-01
BGN Expression Level (log2 TPM)
STAD
10
8
6
4
0.25
0.50
0.75
1.00
0.0
0.1
0.2
0.3
0.4
Purity
Infiltration Level

B) FN1

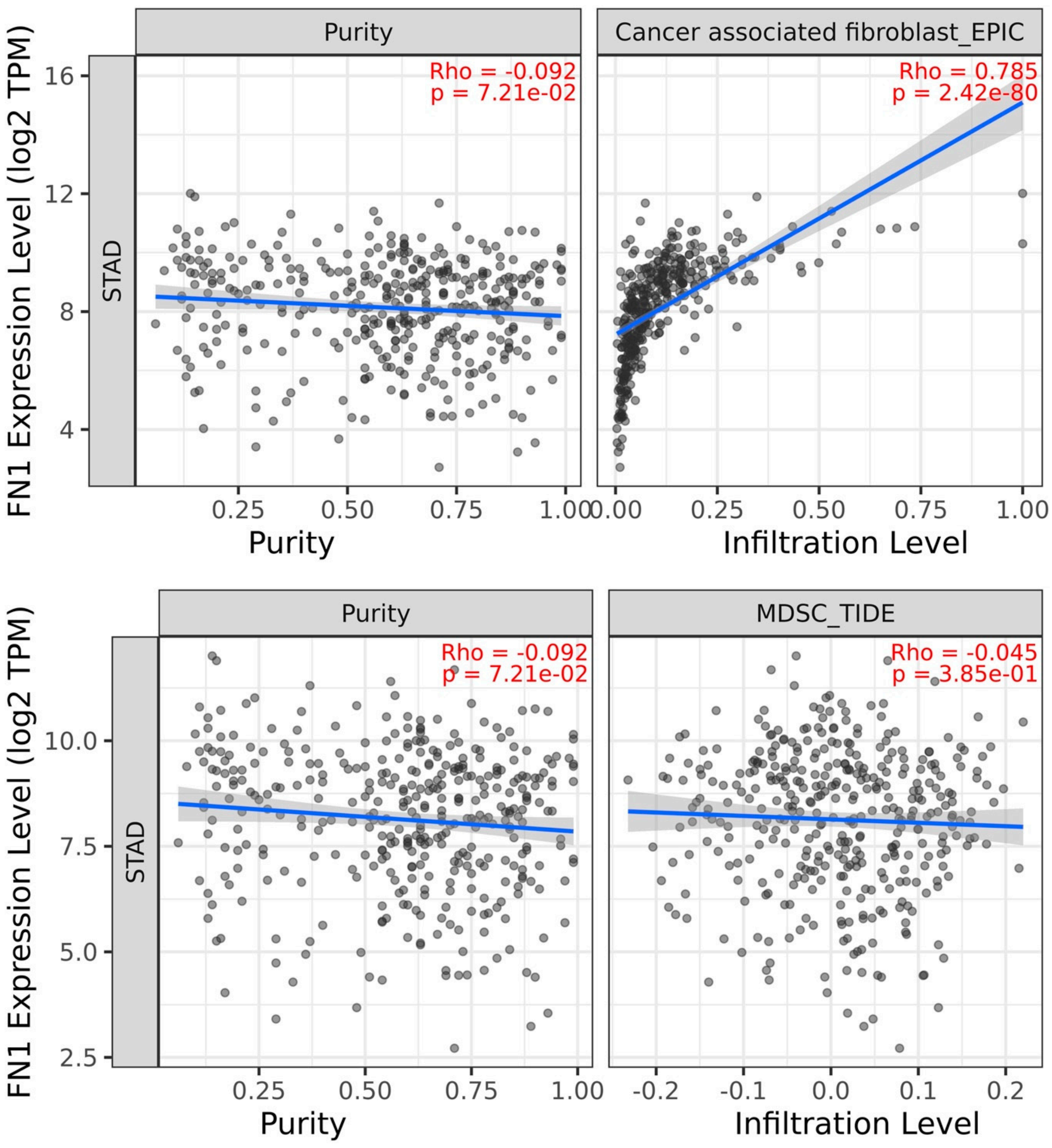

Purity
Cancer associated fibroblast_EPIC
Rho = -0.092
p = 7.21e-02
Rho = 0.785
p = 2.42e-80
STAD
FN1 Expression Level (log2 TPM)
16
12
8
4
0.25
0.50
0.75
1.00
0.00
Purity
Infiltration Level
Purity
MDSC_TIDE
Rho = -0.092
p = 7.21e-02
Rho = -0.045
p = 3.85e-01
10.0
7.5
5.0
2.5
-0.2
-0.1
0.0
0.1
0.2

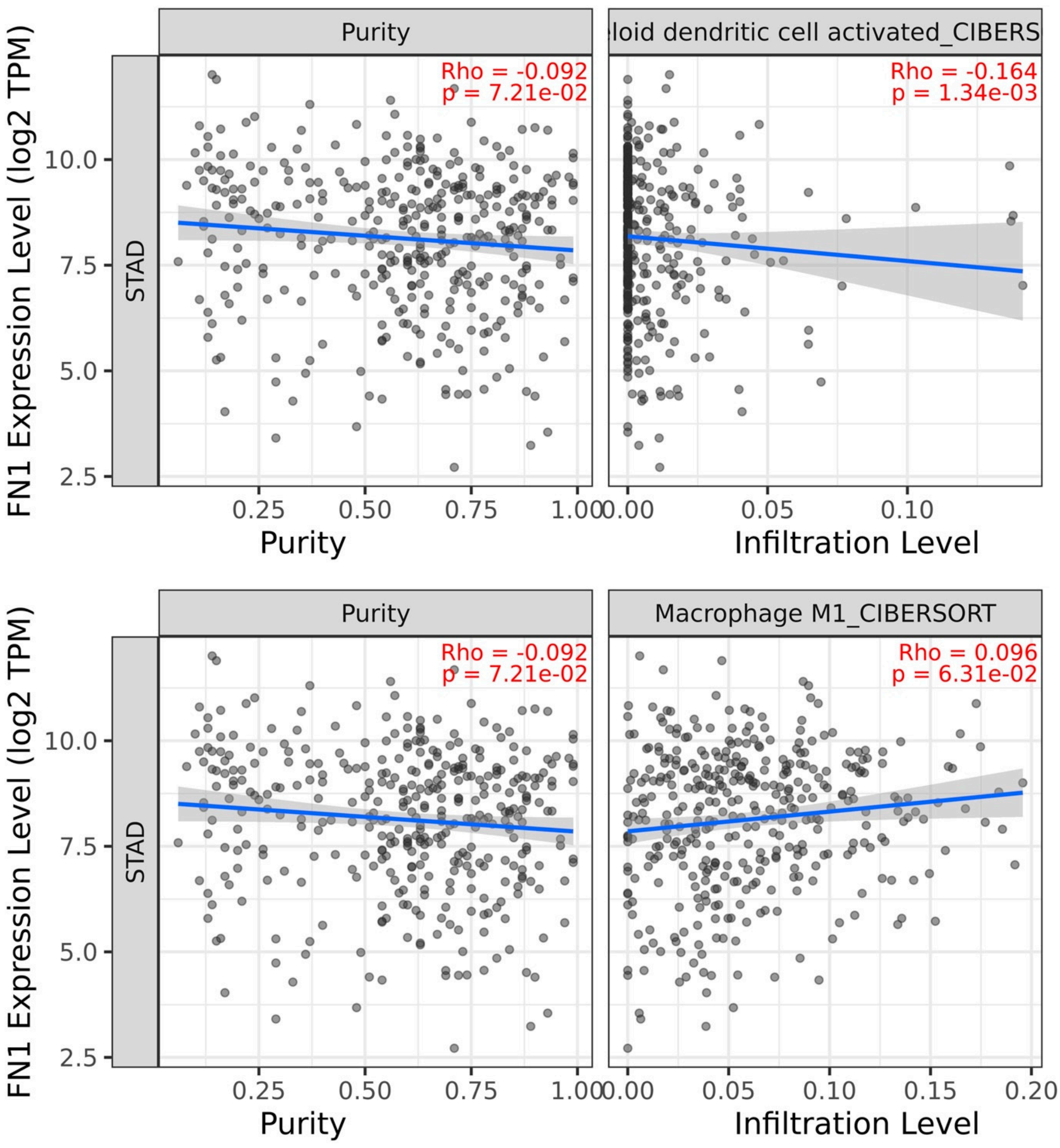

Purity
eloid dendritic cell activated_CIBERS
Rho = -0.092
p = 7.21e-02
Rho = -0.164
p = 1.34e-03
STAD
FN1 Expression Level (log2 TPM)
10.0
7.5
5.0
2.5
0.25
0.50
0.75
1.00
0.00
0.05
0.10
Purity
Infiltration Level
Purity
Macrophage M1_CIBERSORT
Rho = -0.092
p = 7.21e-02
Rho = 0.096
p = 6.31e-02
STAD
FN1 Expression Level (log2 TPM)
10.0
7.5
5.0
2.5
0.25
0.50
0.75
1.00
0.00
0.05
0.10
0.15
0.20
Purity
Infiltration Level

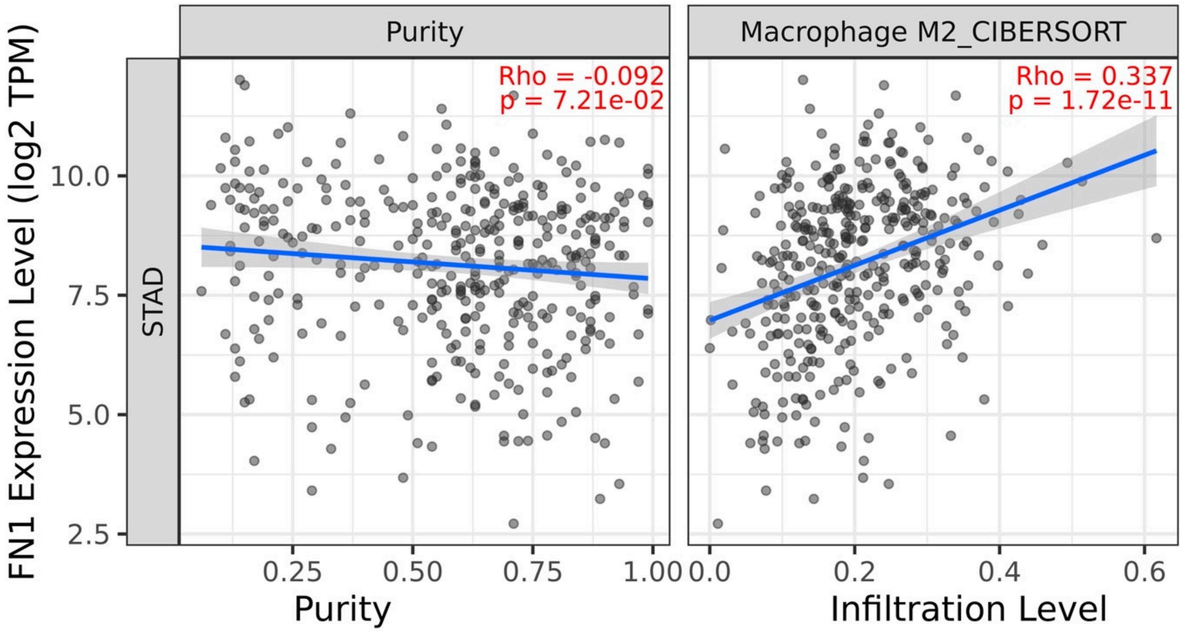
Purity
Macrophage M2_CIBERSORT
Rho = -0.092
p = 7.21e-02
Rho = 0.337
p = 1.72e-11
FN1 Expression Level (log2 TPM)
STAD
10.0
7.5
5.0
2.5
0.25
0.50
0.75
1.00
0.0
0.2
0.4
0.6
Purity
Infiltration Level

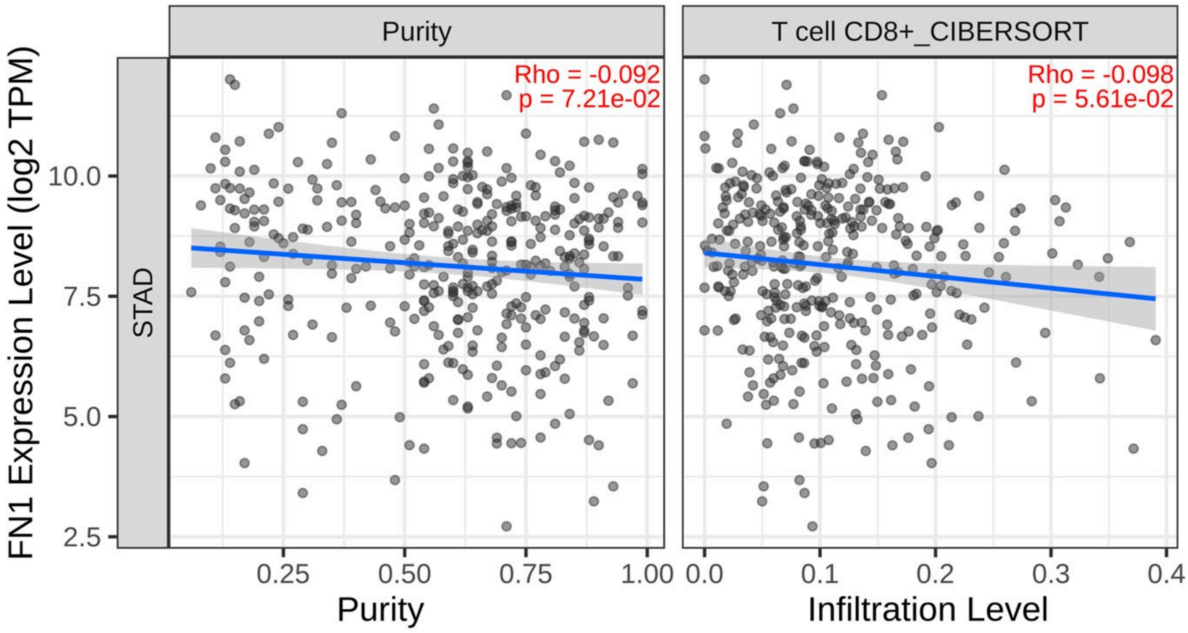
Purity
T cell CD8+_CIBERSORT
Rho = -0.092
p = 7.21e-02
Rho = -0.098
p = 5.61e-02
FN1 Expression Level (log2 TPM)
STAD
10.0
7.5
5.0
2.5
0.25
0.50
0.75
1.00
0.0
0.1
0.2
0.3
0.4
Purity
Infiltration Level

C) THBS2

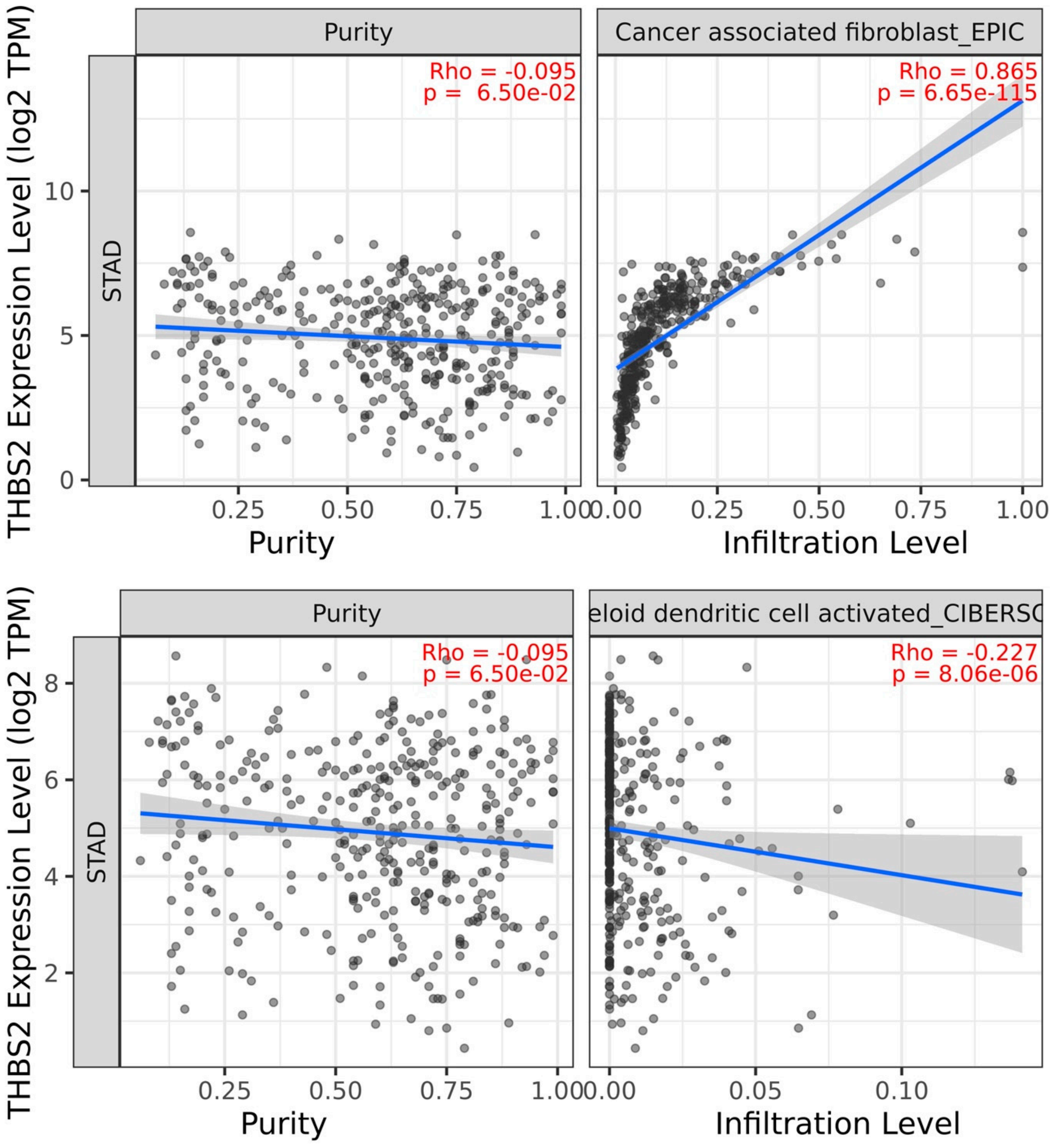

Purity
Cancer associated fibroblast_EPIC
Rho = -0.095
p = 6.50e-02
Rho = 0.865
p = 6.65e-115
STAD
THBS2 Expression Level (log2 TPM)
Purity
Infiltration Level
Purity
eloid dendritic cell activated_CIBERSC
Rho = -0.095
p = 6.50e-02
Rho = -0.227
p = 8.06e-06
STAD
THBS2 Expression Level (log2 TPM)
Purity
Infiltration Level

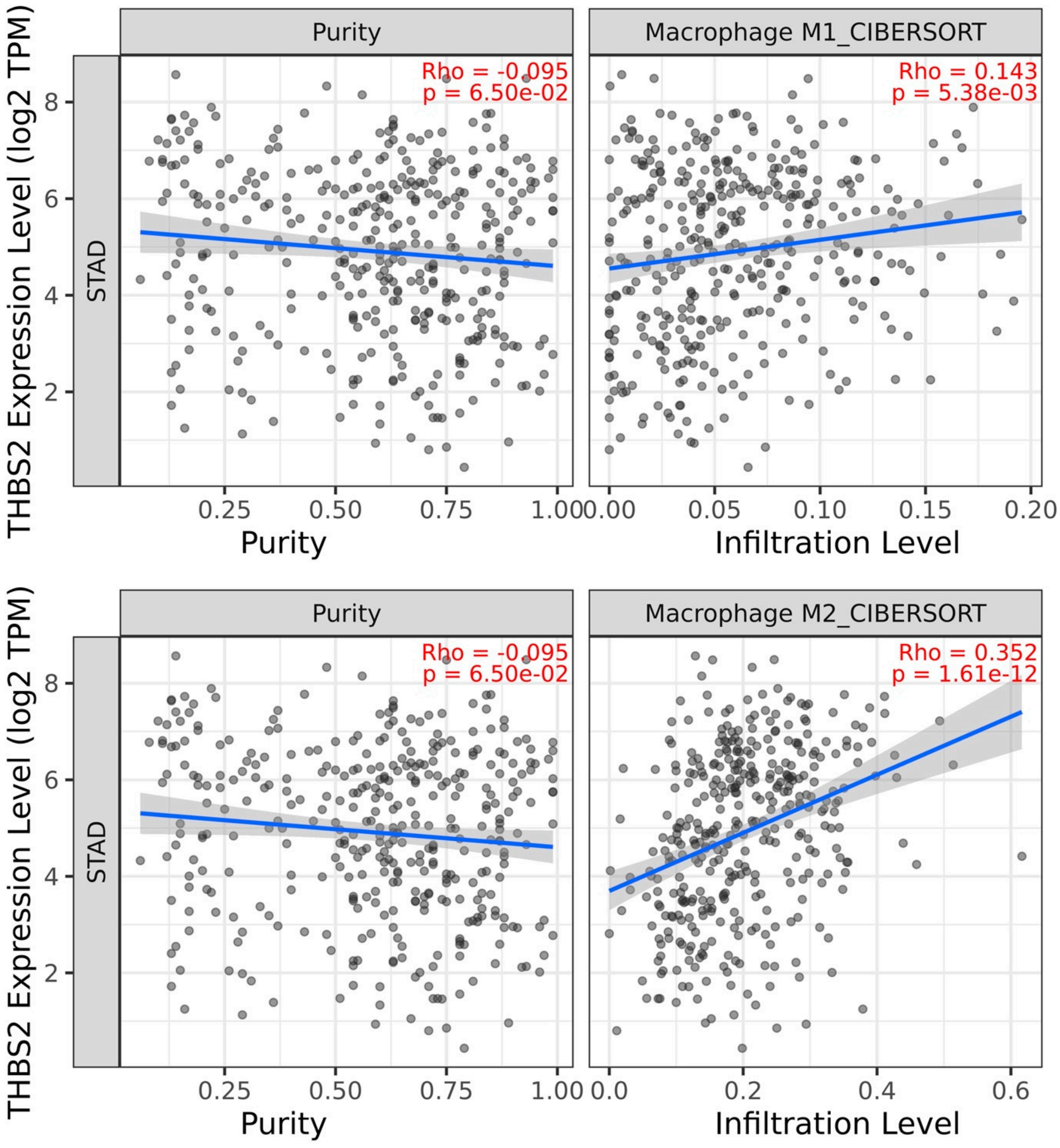

Purity
Macrophage M1_CIBERSORT
Rho = -0.095
p = 6.50e-02
Rho = 0.143
p = 5.38e-03
THBS2 Expression Level (log2 TPM)
STAD
Purity
Infiltration Level
Purity
Macrophage M2_CIBERSORT
Rho = -0.095
p = 6.50e-02
Rho = 0.352
p = 1.61e-12
THBS2 Expression Level (log2 TPM)
STAD
Purity
Infiltration Level

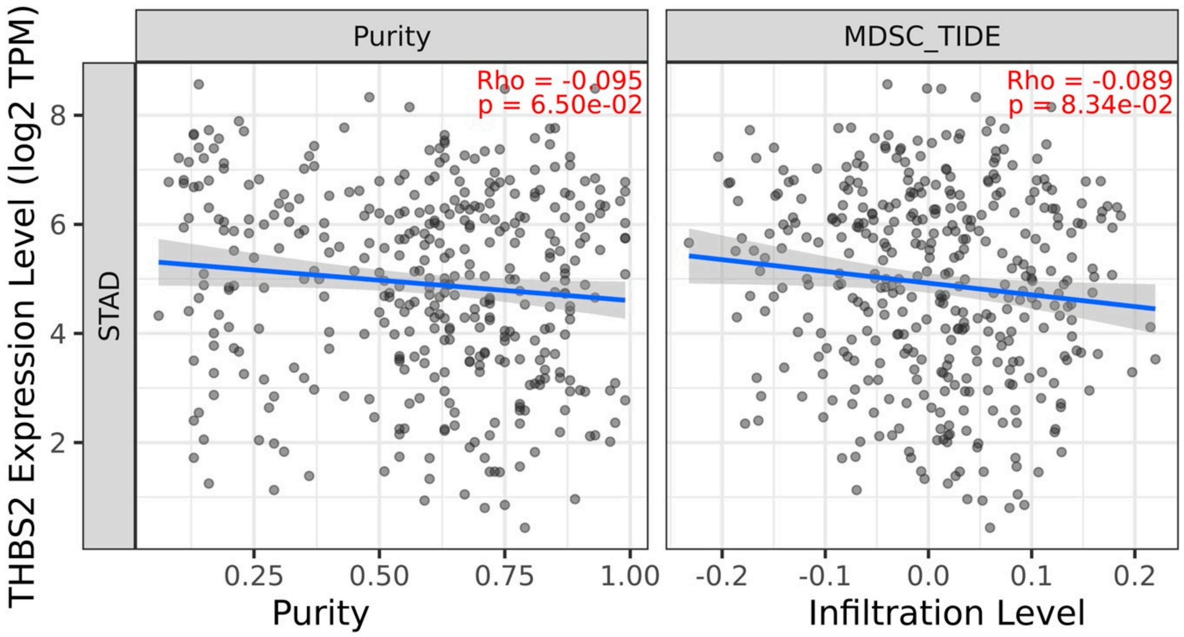
Purity
MDSC_TIDE
Rho = -0.095
p = 6.50e-02
Rho = -0.089
p = 8.34e-02
STAD
THBS2 Expression Level (log2 TPM)
Purity
Infiltration Level

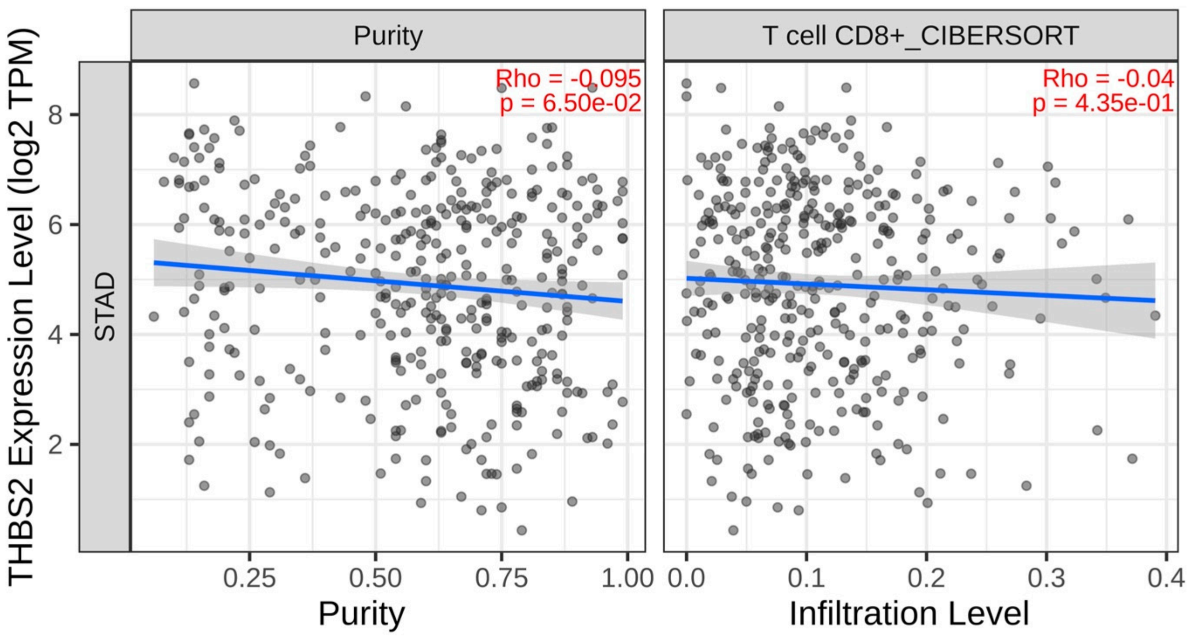
Purity
T cell CD8+_CIBERSORT
Rho = -0.095
p = 6.50e-02
Rho = -0.04
p = 4.35e-01
STAD
THBS2 Expression Level (log2 TPM)
Purity
Infiltration Level

D) COL1A2

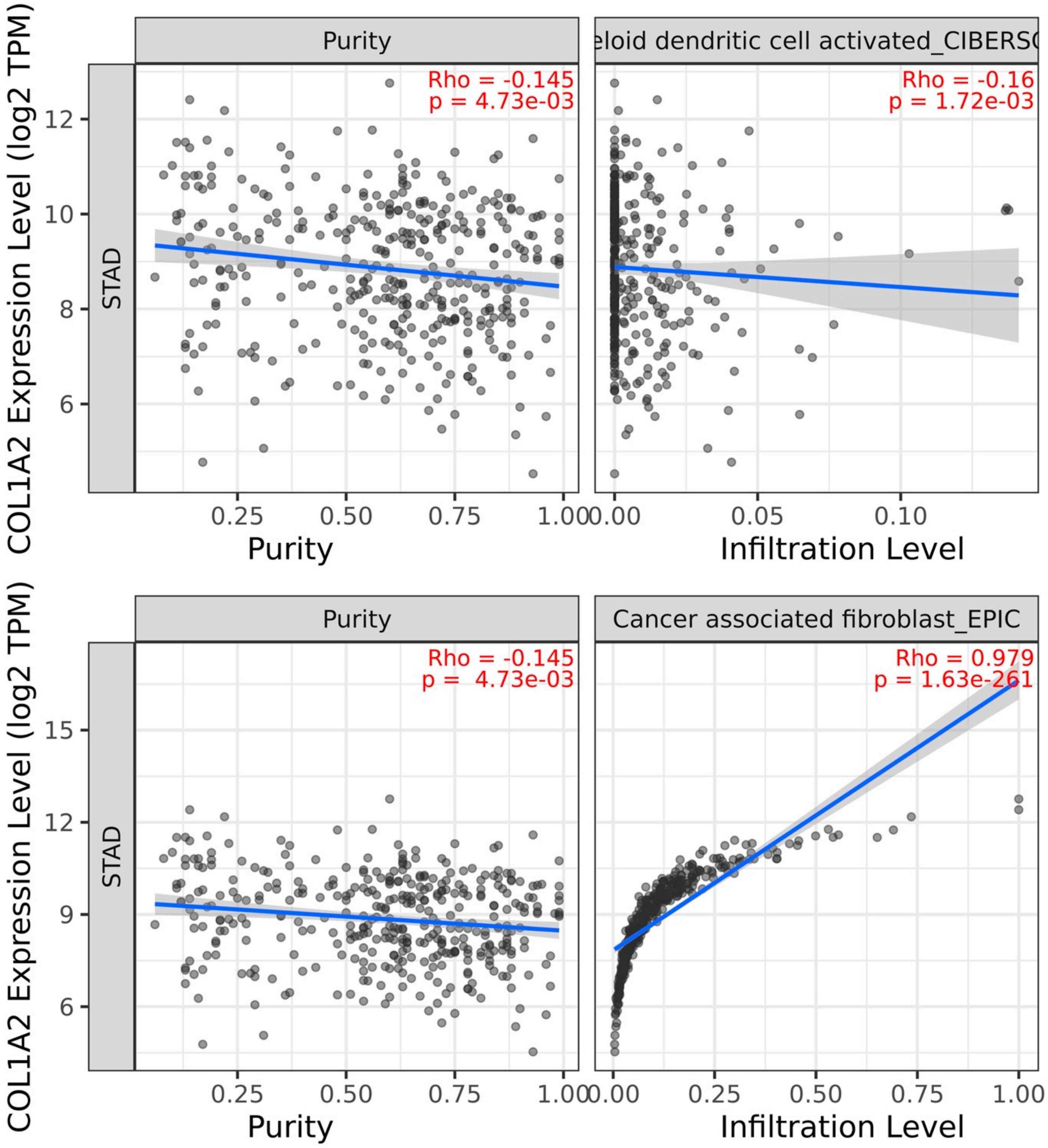

Purity
eloid dendritic cell activated_CIBERSO
STAD
Rho = -0.145
p = 4.73e-03
Rho = -0.16
p = 1.72e-03
COL1A2 Expression Level (log2 TPM)
Purity
Infiltration Level
Purity
Cancer associated fibroblast_EPIC
STAD
Rho = -0.145
p = 4.73e-03
Rho = 0.979
p = 1.63e-261
COL1A2 Expression Level (log2 TPM)
Purity
Infiltration Level

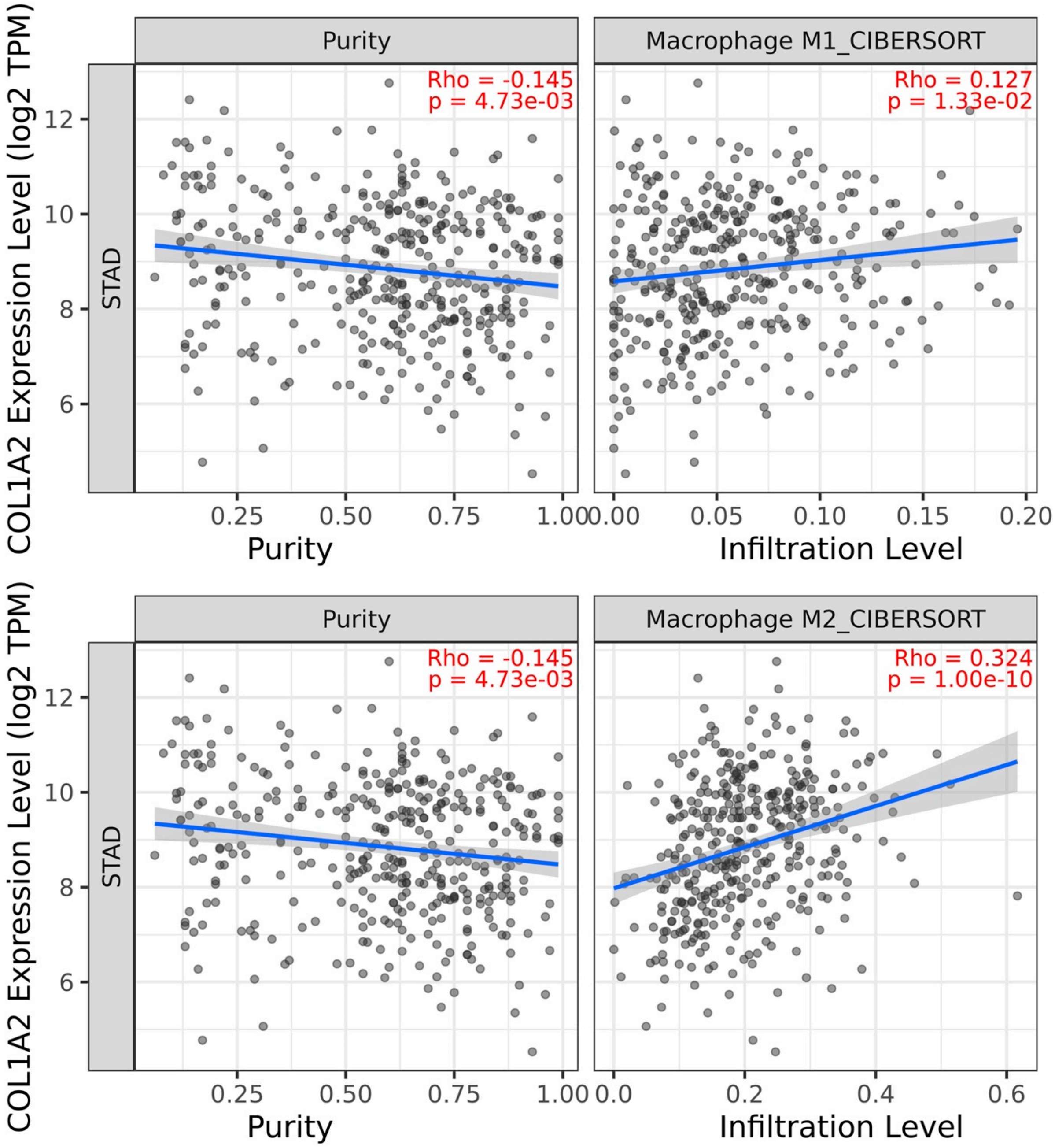
Purity
Macrophage M1_CIBERSORT
Rho = -0.145
p = 4.73e-03
Rho = 0.127
p = 1.33e-02
COL1A2 Expression Level (log2 TPM)
STAD
12
10
8
6
0.25
0.50
0.75
1.00
0.00
0.05
0.10
0.15
0.20
Purity
Infiltration Level
Purity
Macrophage M2_CIBERSORT
Rho = -0.145
p = 4.73e-03
Rho = 0.324
p = 1.00e-10
COL1A2 Expression Level (log2 TPM)
STAD
0.0
0.2
0.4
0.6
Purity
Infiltration Level

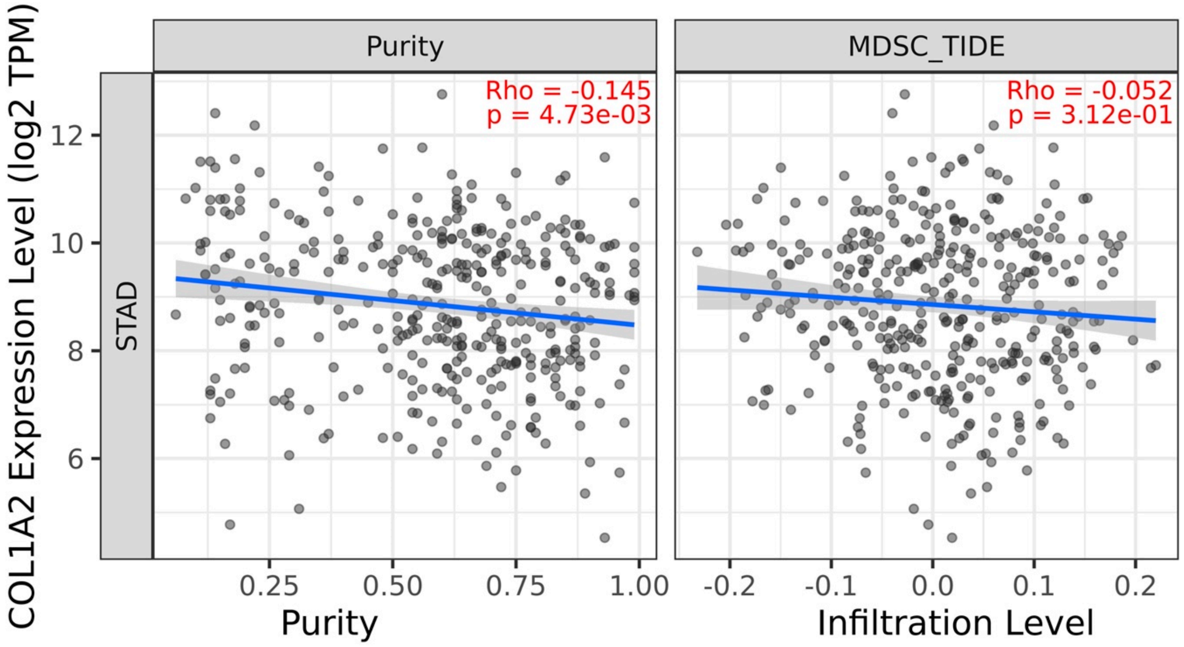

Purity
MDSC_TIDE
Rho = -0.145
p = 4.73e-03
Rho = -0.052
p = 3.12e-01
STAD
COL1A2 Expression Level (log2 TPM)
12
10
8
6
0.25
0.50
0.75
1.00
-0.2
-0.1
0.0
0.1
0.2
Purity
Infiltration Level

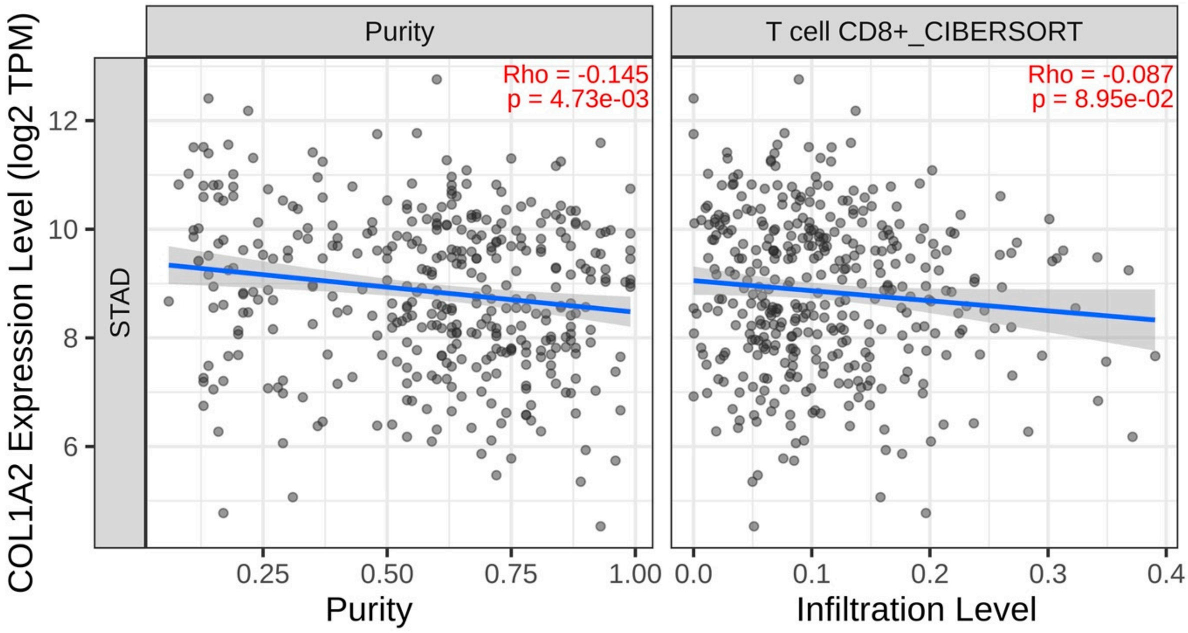

Purity
T cell CD8+_CIBERSORT
Rho = -0.145
p = 4.73e-03
Rho = -0.087
p = 8.95e-02
STAD
COL1A2 Expression Level (log2 TPM)
12
10
8
6
0.25
0.50
0.75
1.00
0.0
0.1
0.2
0.3
0.4
Purity
Infiltration Level

e) COL3A1

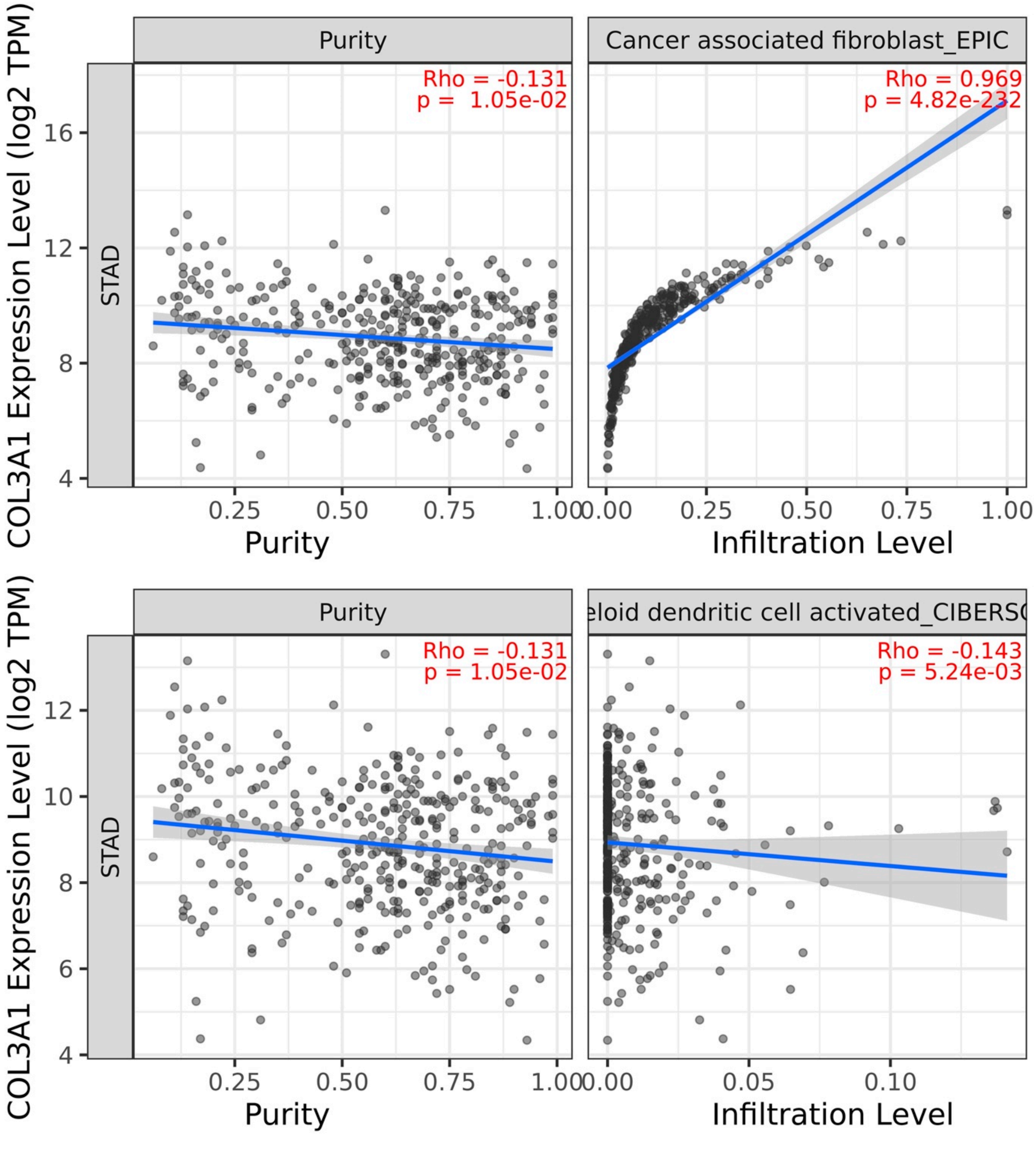

Purity
Cancer associated fibroblast_EPIC
STAD
Rho = -0.131
p = 1.05e-02
Rho = 0.969
p = 4.82e-232
COL3A1 Expression Level (log2 TPM)
Purity
Infiltration Level
Purity
eloid dendritic cell activated_CIBERS(
STAD
Rho = -0.131
p = 1.05e-02
Rho = -0.143
p = 5.24e-03
COL3A1 Expression Level (log2 TPM)
Purity
Infiltration Level

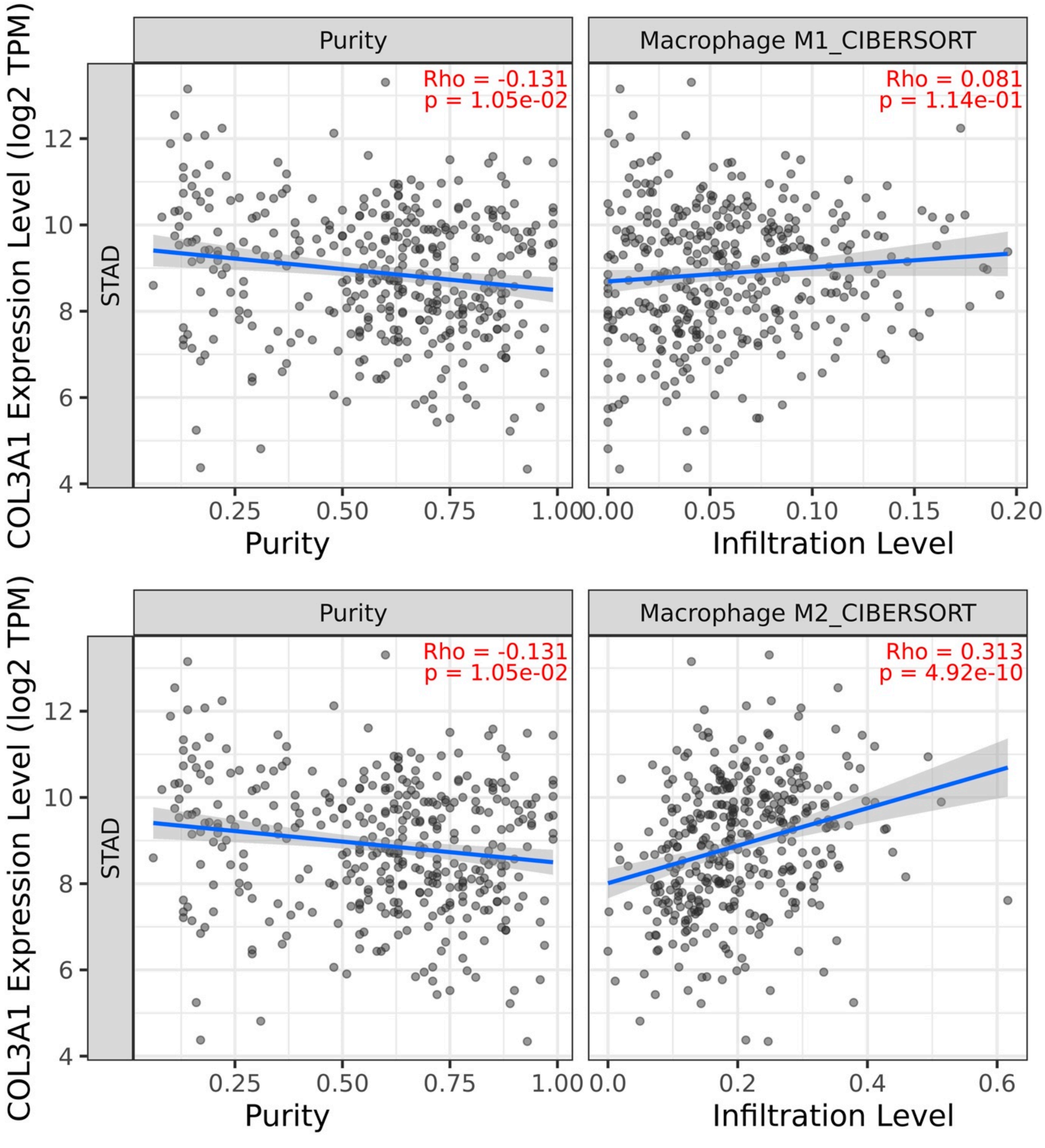

Purity
Macrophage M1_CIBERSORT
Rho = -0.131
p = 1.05e-02
Rho = 0.081
p = 1.14e-01
STAD
COL3A1 Expression Level (log2 TPM)
Purity
Infiltration Level
Purity
Macrophage M2_CIBERSORT
Rho = -0.131
p = 1.05e-02
Rho = 0.313
p = 4.92e-10
STAD
COL3A1 Expression Level (log2 TPM)
Purity
Infiltration Level

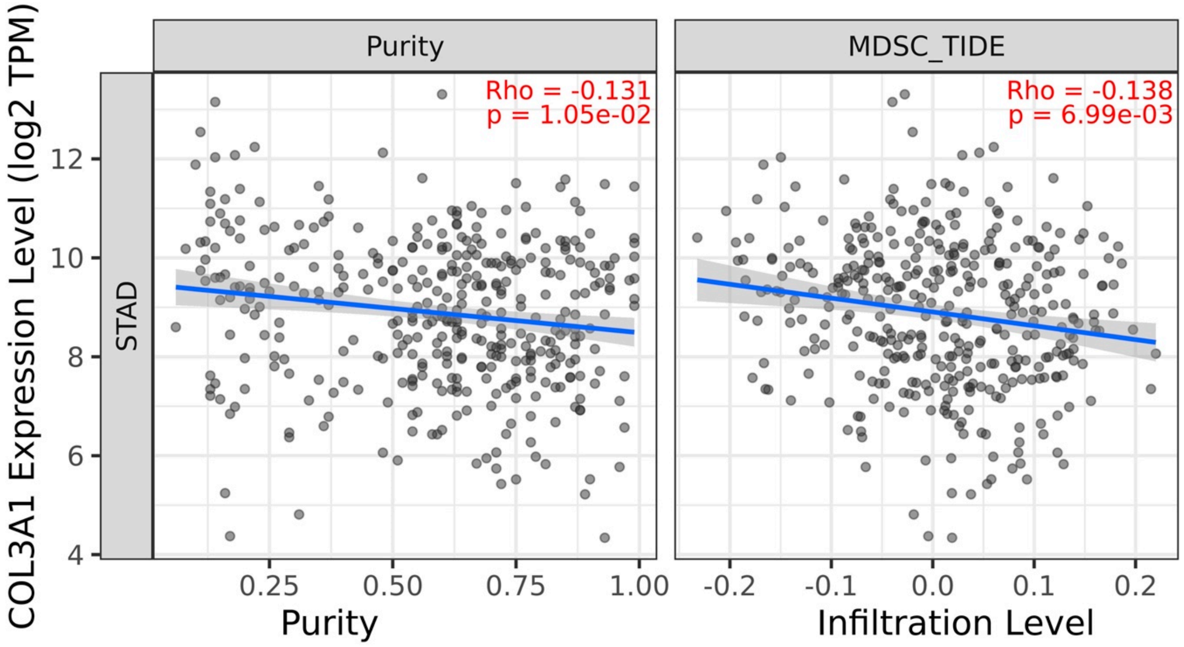
Purity
MDSC_TIDE
Rho = -0.131
p = 1.05e-02
Rho = -0.138
p = 6.99e-03
COL3A1 Expression Level (log2 TPM)
STAD
Purity
Infiltration Level

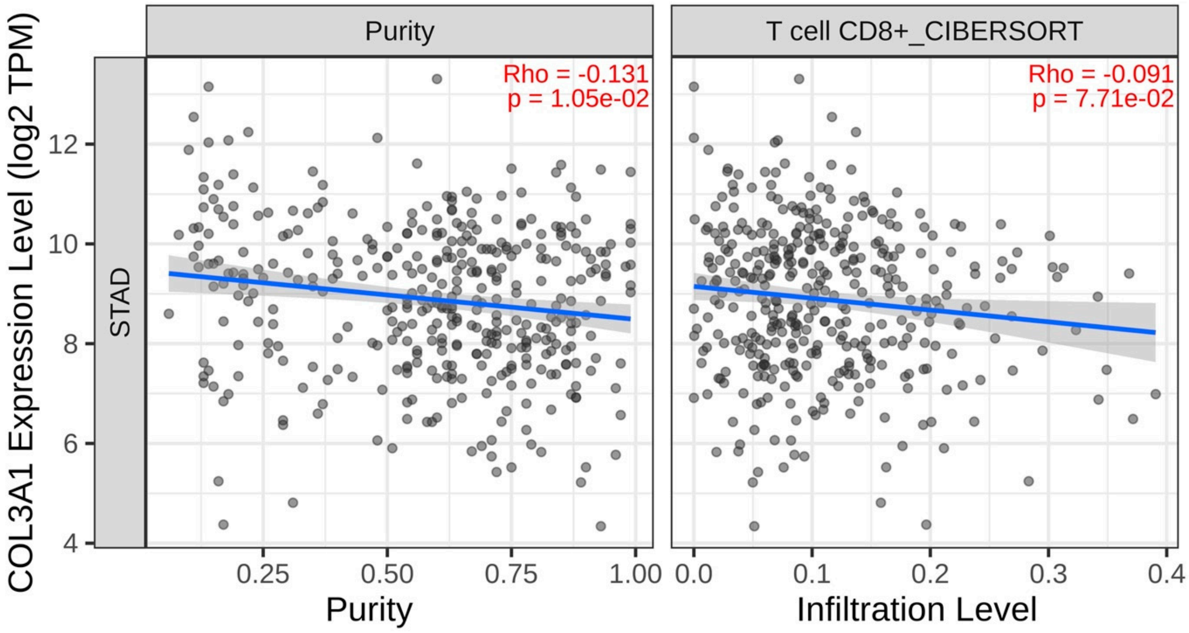
Purity
T cell CD8+_CIBERSORT
Rho = -0.131
p = 1.05e-02
Rho = -0.091
p = 7.71e-02
COL3A1 Expression Level (log2 TPM)
STAD
Purity
Infiltration Level

f) COL5A1

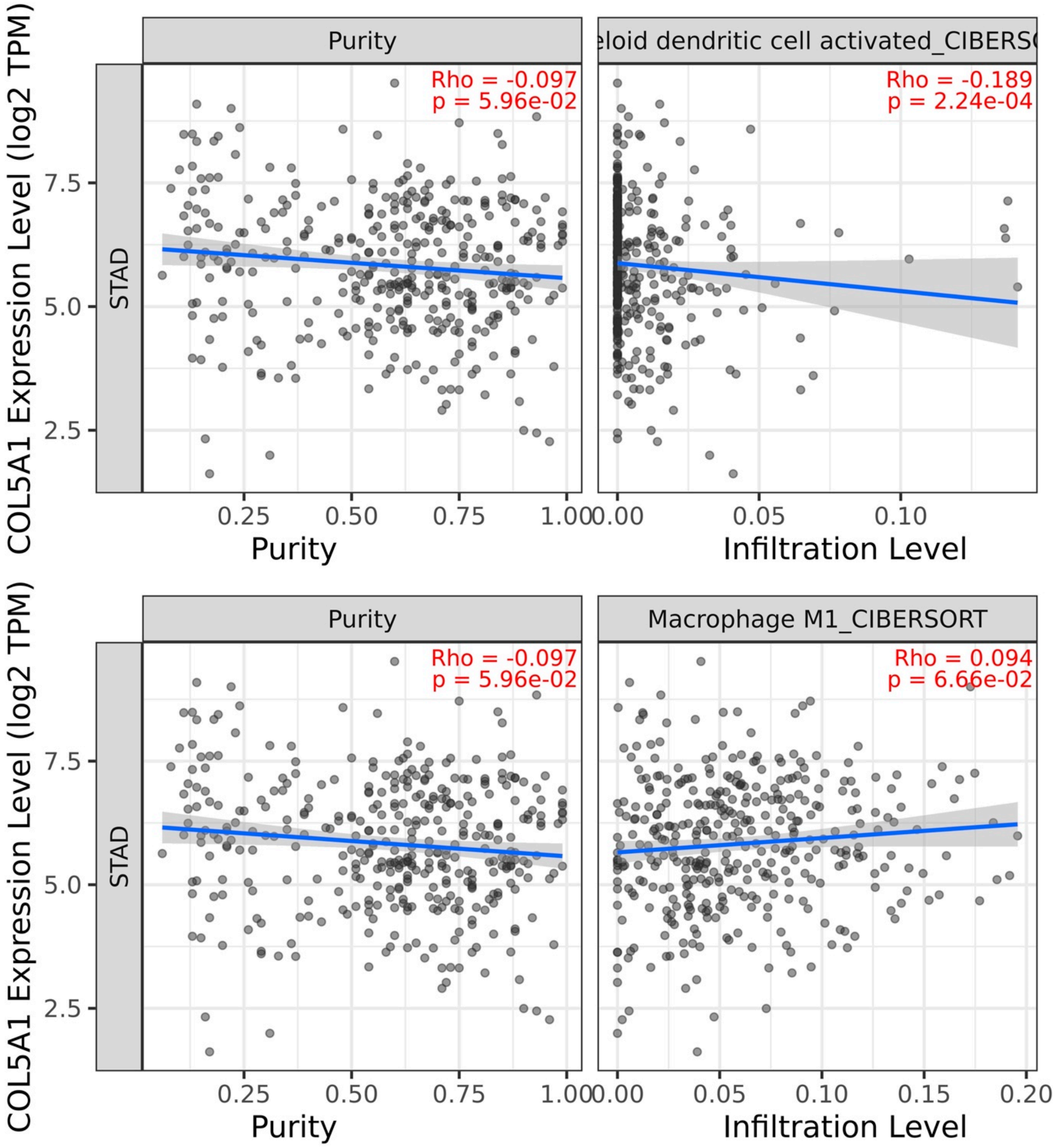

COL5A1 Expression Level (log2 TPM)
Purity
eloid dendritic cell activated_CIBERS(
STAD
Rho = -0.097
p = 5.96e-02
Rho = -0.189
p = 2.24e-04
7.5
5.0
2.5
0.25
0.50
0.75
1.00
0.00
0.05
0.10
Purity
Infiltration Level
COL5A1 Expression Level (log2 TPM)
Purity
Macrophage M1_CIBERSORT
STAD
Rho = -0.097
p = 5.96e-02
Rho = 0.094
p = 6.66e-02
7.5
5.0
2.5
0.25
0.50
0.75
1.00
0.00
0.05
0.10
0.15
0.20
Purity
Infiltration Level

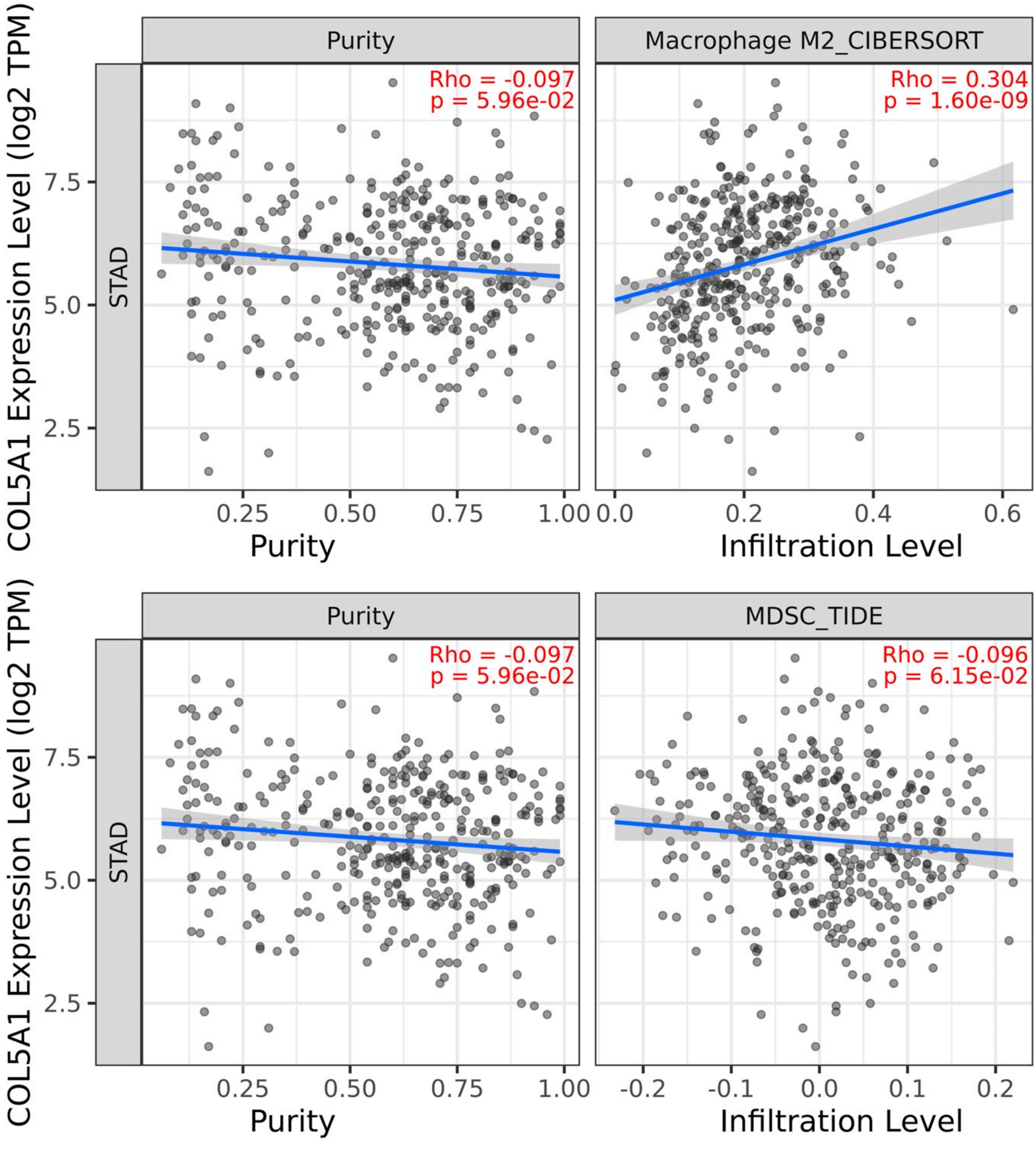
Purity
Macrophage M2_CIBERSORT
Rho = -0.097
p = 5.96e-02
Rho = 0.304
p = 1.60e-09
STAD
COL5A1 Expression Level (log2 TPM)
7.5
5.0
2.5
0.25
0.50
0.75
1.00
0.0
0.2
0.4
0.6
Purity
Infiltration Level
Purity
MDSC_TIDE
Rho = -0.097
p = 5.96e-02
Rho = -0.096
p = 6.15e-02
STAD
COL5A1 Expression Level (log2 TPM)
7.5
5.0
2.5
0.25
0.50
0.75
1.00
-0.2
-0.1
0.0
0.1
0.2
Purity
Infiltration Level

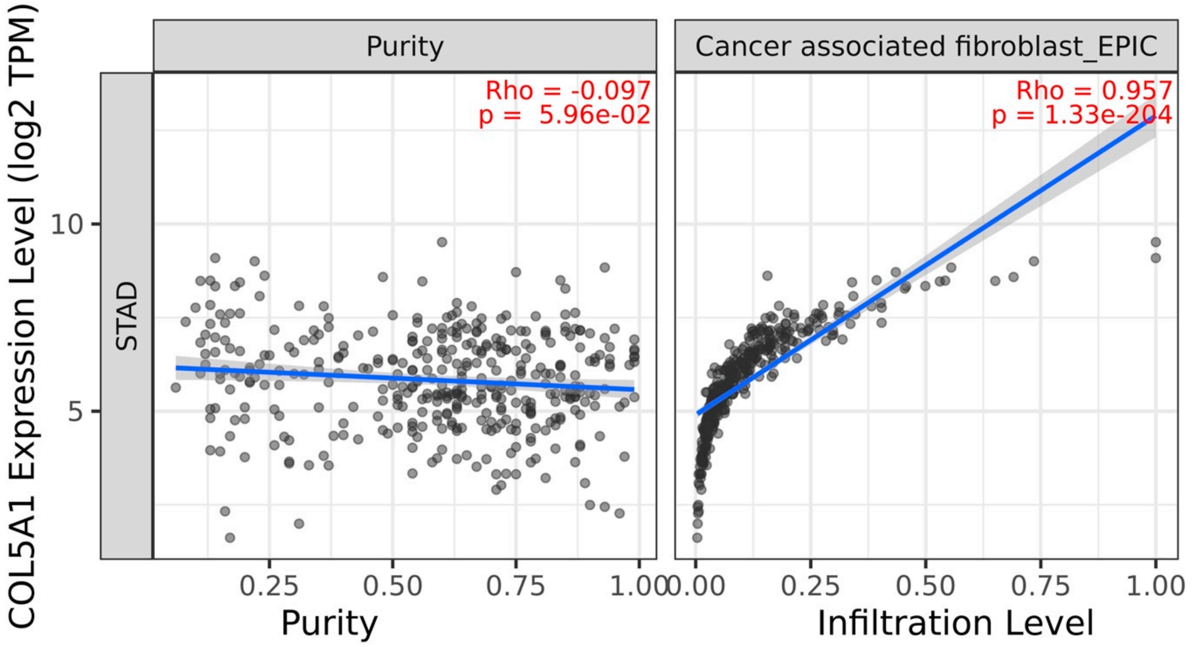
Purity
Cancer associated fibroblast_EPIC
Rho = -0.097
p = 5.96e-02
Rho = 0.957
p = 1.33e-204
STAD
COL5A1 Expression Level (log2 TPM)
10
5
0.25
0.50
0.75
1.00
0.00
0.25
0.50
0.75
1.00
Purity
Infiltration Level

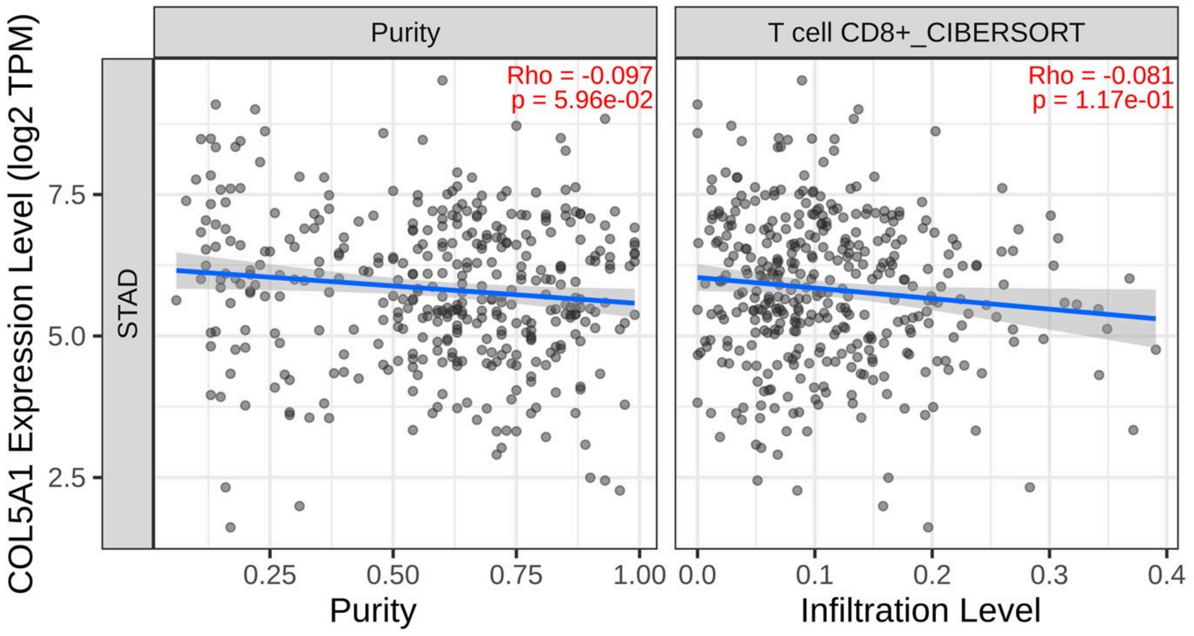
Purity
T cell CD8+_CIBERSORT
Rho = -0.097
p = 5.96e-02
Rho = -0.081
p = 1.17e-01
STAD
COL5A1 Expression Level (log2 TPM)
7.5
5.0
2.5
0.25
0.50
0.75
1.00
0.0
0.1
0.2
0.3
0.4
Purity
Infiltration Level
